# Spatial migration of human reward processing with functional development: Evidence from quantitative meta‐analyses

**DOI:** 10.1002/hbm.25103

**Published:** 2020-07-07

**Authors:** Zachary A Yaple, Rongjun Yu, Marie Arsalidou

**Affiliations:** ^1^ Department of Psychology National University of Singapore Singapore Singapore; ^2^ NUS Graduate School for Integrative Sciences and Engineering National University of Singapore Singapore Singapore; ^3^ Department of Psychology National Research University Higher School of Economics Moscow Russian Federation; ^4^ Department of Psychology, Faculty of Health York University Toronto Canada

**Keywords:** adolescents, children, developmental neuroscience, fMRI meta‐analysis, reward processing

## Abstract

Functional magnetic resonance imaging (fMRI) studies have shown notable age‐dependent differences in reward processing. We analyzed data from a total of 554 children, 1,059 adolescents, and 1,831 adults from 70 articles. Quantitative meta‐analyses results show that adults engage an extended set of regions that include anterior and posterior cingulate gyri, insula, basal ganglia, and thalamus. Adolescents engage the posterior cingulate and middle frontal gyri as well as the insula and amygdala, whereas children show concordance in right insula and striatal regions almost exclusively. Our data support the notion of reorganization of function over childhood and adolescence and may inform current hypotheses relating to decision‐making across age.

## Graphical abstract

For reward processing, adults engage an extended set of regions that include anterior and posterior cingulate gyri, insula, basal ganglia, and thalamus. Adolescents engage the posterior cingulate and middle frontal gyri as well as the insula and amygdala. Children show concordance in right insula and striatal regions almost exclusively.

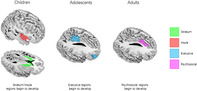

## 1 BACKGROUND

Etymologically, “reward” derives from Old French “regard” and corresponds to a prize or wage in exchange for some service. Humans experience reward regularly, a process that encompasses a broad range of decision‐making phenomena including risky decision‐making (Mohr, Biele, & Heekeren, 2010; Yaple, Martinez‐Saito, Feurra, Shestakova, & Klucharev, 2017; Yu & Zhou, 2009), delay discounting (McClure, Laibson, Loewenstein, & Cohen, 2004; Wesley & Bickel, 2014), feedback learning (Luft, 2014; Yaple et al., 2018) and reward anticipation (Knutson & Greer, 2008; Oldham et al., 2018).

Neuroscientists identified a set of brain areas associated with reward processing that include subcortical (e.g., nucleus accumbens, caudate, putamen, thalamus) and cortical regions (e.g., insula, prefrontal, and cingulate cortices; Knutson et al., 2001, b; O'Doherty, Kringelbach, Rolls, Hornak, & Andrews, 2001; Knutson & Cooper, 2005; Kuhnen & Knutson, 2005; Balleine, Delgado, & Hikosaka, 2007; Rao, Korczykowski, Pluta, Hoang, & Detre, 2008; Fujiwara, Tobler, Taira, Iijima, & Tsutsui, 2009; Mohr et al., 2010; Liu, Hairston, Schrier, & Fan, 2011; Diekhof, Kaps, Falkai, & Gruber, 2012; Kohls et al., 2013; Silverman, Jedd, & Luciana, 2015). A sufficient number of articles have accumulated, resulting in a series of meta‐analyses on reward processing in adults (Arsalidou, Vijayarajah, & Sharaev, in press; Sescousse et al, 2013). These studies revealed a distributed set of active areas in the prefrontal, insular, and anterior cingulate cortices, as well as striatum, amygdala, and thalamus.

Brain representations of reward processing have been examined using functional neuroimaging in children and adults. Children typically select risky options more often than adults in tasks that require selections between a gamble and a sure option (Harbaugh, Krause, & Vesterlund, 2002; Levin & Hart, 2003; Levin, Hart, Weller, & Harshman, 2007; Paulsen, Carter, Platt, Huettel, & Brannon, 2012; Paulsen, Platt, Huettel, & Brannon, 2011; Rakow & B Rahim, 2010; Weller, Levin, & Denburg, 2011). Likewise, the tendency to prefer larger delayed rewards improves with age, since children tend to favor immediate rewards more often than adults (Banich et al., 2013; Ellis et al., 2012; Green, Fry, & Myerson, 1994; Green, Myerson, & Ostaszewski, 1999; Prencipe et al., 2011; Thompson, Barresi, & Moore, 1997). Unlike adults and adolescents, children are less capable of integrating previous encounters when deciding to select between risk and safe options (Paulsen et al., 2012). For instance, when comparing children with adults performing gambling tasks, children tend to perform worse (Bechara, Damasio, Damasio, & Anderson, 1994; Crone, Bunge, Latenstein, & van der Molen, 2005; Crone & van der Molen, 2004; Garon & Moore, 2004; Kerr & Zelazo, 2004; Prencipe et al., 2011); however, their performance is much better than adults when the memory component is simplified (Brainerd, 1981; Garon & Moore, 2007). Consistent with this premise, research finds that children (8–12 years) are unable to form model‐based strategies as compared to adolescents (13–17 years) and adults (18–25 years; Decker, Otto, Daw, & Hartley, 2016; Potter et al., 2017). One possibility may be that children lack the resources required for making reward‐related decisions due to limitations in their mental‐attentional capacity (e.g., Arsalidou & Im‐Bolter, 2017; Arsalidou & Pascual‐Leone, 2016; Arsalidou, Pascual‐Leone, & Johnson, 2010; Pascual‐Leone, 1970). In contrast, adults and adolescents may rely on a similar set of brain regions when performing reward‐related tasks involving risky decision‐making, delay discounting, feedback processing and reward anticipation. Adolescents often show hyperactivation of various regions compared to adults (Christakou, Brammer, & Rubia, 2011; de Macks et al., 2011; Forbes et al., 2010; Galvan et al., 2006; Jarcho et al., 2012; Paulsen et al., 2012; Ripke et al., 2012; van Duijvenvoorde et al., 2014), with other studies reporting cases of *reduced* reward‐anticipatory activation via striatum activity in adolescence (Bjork, Chen, Smith, & Hommer, 2010; Bjork, Knutson, & Hommer, 2008; Bjork, Smith, Chen, & Hommer, 2010; Lamm et al., 2014; Peters et al., 2011).

A common issue with empirical findings is that studies comparing brain responses across different age groups are oftentimes inconsistent (Richards, Plate, & Ernst, 2013). For example, some show suprathreshold activity in the basal ganglia for children, and no suprathreshold activity in superior/medial frontal gyri for adults when they process rewards (Kappel et al., 2013), whereas, others have demonstrated less basal ganglia activity and more medial frontal cortex activity in late compared with early adolescence (e.g., Forbes et al., 2010). Heterogeneity of task design and complexity of the behaviors being studied have been identified as explanations for such inconsistencies (Richards et al., 2013 for review). Richards et al. (2013) also emphasize that the developing brain is a moving target, meaning that different systems of regions may develop with different trajectories (Giedd, 2004). For instance, the amygdala, hippocampus and insula are implicated in aversive behaviors and appear to follow a quadratic developmental trajectory (Arnett, 1999; Larson, Moneta, Richards, & Wilson, 2002; Silk, Steinberg, & Morris, 2003; Weinstein, Mermelstein, Hankin, Hedeker, & Flay, 2007), whereas executive regions such as the anterior cingulate and prefrontal cortex operate as an executive regulation subsystem (Haber & Knutson, 2010) that develops linearly with age (Casey, Jones, & Hare, 2008; Li, 2017; Marsh et al., 2006; Rubia et al., 2006; Rubia, Smith, Taylor, & Brammer, 2007). In summary, different developmental trajectories may explain variability among empirical findings.

Reward‐related studies have proposed top‐down modulation of subcortical regions via direct corticostriatal projections (Haber & Knutson, 2010), whereas others have demonstrated indirect modulation of the ventromedial prefrontal cortical regions (coding for valuation) by the executive subsystem within the dorsolateral prefrontal cortex (coding for self‐control; Hare et al., 2009). This framework is consistent with studies revealing an increase in reward sensitivity and risky decision making during adolescence (Schneider et al., 2012; van Duijvenvoorde et al., 2014; van Leijenhorst et al., 2010). Perhaps reward‐processing during adolescence may be the result of an executive system that is still under development (Prencipe et al., 2011; Steinbeis, Haushofer, Fehr, & Singer, 2014; Steinberg et al., 2009); an executive system that fails to maintain the balance between an overcompensating striatum and a diminished insula (Ernst & Fudge, 2009).

Meta‐analytic approaches on age offer a quantitative approach for addressing these types of examinations (e.g., Yaple and Yu, 2020). Activation likelihood estimation (ALE), for example, evaluates concordance of brain coordinates reported across functional magnetic resonance imaging (fMRI) studies. Past ALE meta‐analyses show that adolescents compared with adults exhibit greater concordance within the insula, ventral and dorsal striatum, amygdala and anterior and posterior and anterior cingulate cortex (Silverman et al., 2015; also see Bartra, McGuire, & Kable, 2013). The authors attributed their results to specific cognitive mechanisms associated with higher reward seeking behaviors in adolescents, since adults showed no activation greater than adolescents. However, many studies included participants that were younger than 13 years in the adolescent group (Forbes et al., 2010; Schlund et al., 2010; van Leijenhorst, Crone, & Bunge, 2006; van Leijenhorst, Moor, et al., 2010; van Leijenhorst, Zanolie, et al., 2010) or included both children and adolescents (Christakou et al., 2011; Ernst et al., 2005; Jarcho et al., 2012; May et al., 2004). A comprehensive meta‐analysis of functional brain correlates of children performing reward tasks has not yet been conducted and estimates of conjunction and disjunction of brain responses to rewards among children, adolescents, and adults are lacking.

In order to investigate concordance of brain correlates across studies and find overarching patterns in the literature we perform a series of quantitative ALE meta‐analyses across data derived from children, adolescents and adults. We first examine data associated with general reward processing, to identify regions that engage in all reward‐related functions. Based on the notion that the executive control system is still under development during adolescence, we expected our fMRI meta‐analyses to reveal greater prefrontal and cingulate activity across studies for adults compared to adolescents and children, and no intact executive system in children. To further explore the role of each of the regions in the reward network, we also performed supplementary analyses on (a) experiments related only to reward outcomes, (b) experiments related only to reward anticipation, and (c) experiments related only to the monetary incentive delay task. Finally, we explore the prevalence of reported executive regions across the literature in order to assess the pivotal stages of development for these regions.

## 2 METHODS

### 2.1 Literature search and article selection

Eligible studies were recovered from past meta‐analyses of adults (e.g., Sescousse et al., 2013; *n* = 22 eligible) and adolescents (Silverman et al., 2015; *n* = 6 eligible studies). Literature from subsequent years was searched using the Web of Science database (http://www.webofknowledge.com). Due to the vast number of reward‐related studies in adults, we performed three independent searches using keywords: (a) “reward” AND “youth”, (b) “reward” AND “children,” and (c) “reward” AND “adolescents.” These searches were combined and after removing 174 duplicates, 490 articles were screened for eligibility. For adults, in addition to the 22 eligible studies from Sescousse et al. (2013) we performed a search from 2013 to 2018 with the keywords: [“fMRI” OR “neuroimaging”] AND [“money” OR “monetary” OR “financial”] AND “reward”, yielding a total of 499 articles.

Eligible articles included reward‐related contrasts (e.g., reward anticipation, reward outcome, positive vs. negative feedback, etc.) to correspond with previous fMRI meta‐analyses on reward processing (Diekhof et al., 2008; Mohr et al., 2010; Liu et al., 2011; Diekhof et al., 2012; Sacchet and Knutson, 2013; Sescousse et al., 2013; Montoya et al., 2014; Morellia et al., 2015; Wesley & Bickel, 2014; Silverman et al., 2015; Oldham et al., 2018). Exclusion criteria include articles that did not report whole brain fMRI coordinates in MNI or Talairach space, articles that did not report reward‐related contrast associated with risky decision‐making, delay discounting or feedback learning, and articles that did not report healthy human volunteers within specified mean ages for the following age groups: children (between 6 and 12.9 years), adolescents (13 and 17.9 years), and young adults (18 and 35 years). See Supplemental Tabes S1–S3 for the list of eligible articles included in the meta‐analyses. Figures S1 and S2 for flowcharts showing the yield of the searches and the steps taken to screen and identify eligible articles for children/adolescents and adults.

Two authors independently selected articles meeting these criteria, and final decisions were made in agreement. The final dataset contained data from 18 eligible articles (28 experiments) for children, 29 articles (46 experiments) for adolescents, and 70 articles (90 experiments) for adults. Because our main between‐group variable was age, we excluded studies that tested groups with large age‐ranges (e.g., 18–70 years). Participant groups and foci included in the three meta‐analyses were exclusive.

Several articles reported more than one relevant experiment, all of which were included in the analyses to improve statistical power, as the latest and currently recommended ALE analysis algorithm accounts for within‐group effects (Turkeltaub et al., 2002). Three main meta‐analyses were performed: (a) children (18 articles; 28 experiments; 19 subject groups; 211 foci), (b) adolescents (29 articles; 46 experiments; 32 subject groups; 586 foci), and (c) young adults (70 articles; 90 experiments; 70 subject groups; 1,010 foci) all of which satisfy current ALE power recommendations of including a minimum of 17 experiments (Eickhoff et al., 2017). Reward sub‐categories associated specifically with reward anticipation did not fulfill the criterion of a minimum of 17 experiments for all age groups, therefore related results are reported in supplementary material. We also performed contrast analyses and computed conjunctions and differences among age groups.

### 2.2 Software and analysis

We analyzed data coordinates using GingerALE, which is a freely available, quantitative meta‐analysis method. This method was first proposed by Turkeltaub et al. (2002), with the latest version described by Eickhoff et al. (2009, 2017). GingerALE (version 2.3.6) was used, which relies on ALE (http://brainmap.org/ale/). ALE compares foci from multiple articles and estimates the magnitude of overlap between foci, yielding clusters most likely to become active across studies. The most recent algorithm minimizes within‐group effects and provides increased power by allowing for inclusion across all possible contrasts (Eickhoff et al., 2017; Turkeltaub et al., 2012). All coordinates were transformed into a common atlas space (Talairach) using the Lohrenz, McCabe, Camerer, and Montague (2007) transformation algorithm. Resulting statistical maps were thresholded at *p* < .05 using a cluster‐level correction for multiple comparisons and a cluster forming threshold at *p* < .001 (Eickhoff et al., 2017). Contrast and conjunction analyses were also performed to compare differences and overlap across age groups, respectively. The threshold for group‐contrasts was set to *p* < .01 uncorrected for multiple comparisons (5,000 permutations, 50 mm^3^ minimum cluster‐size; e.g., Arsalidou et al., 2018), because group‐contrast analyses use cluster‐level thresholded ALE maps for each group, which have already been controlled for multiple comparisons.

## 3 RESULTS

Data from a total of 3,444 participants were used for this study. Participant sample size and mean ages (± SD) in our resulting groups were 554 children with a mean age of 10.80 ± 1.48 (range: 6.9–12.5) years, 1,059 adolescent participants with a mean age of 14.82 ± 0.96 (range: 13.39–17.1), and 1,831 young adults with a mean age of 24.38 ± 2.52 (range: 19.6–29.9) years. Participants for each meta‐analysis were 44.18, 59.53, and 55.45% male for children, adolescents and young adults, respectively.

### 3.1 ALE maps

**FIGURE 1:**
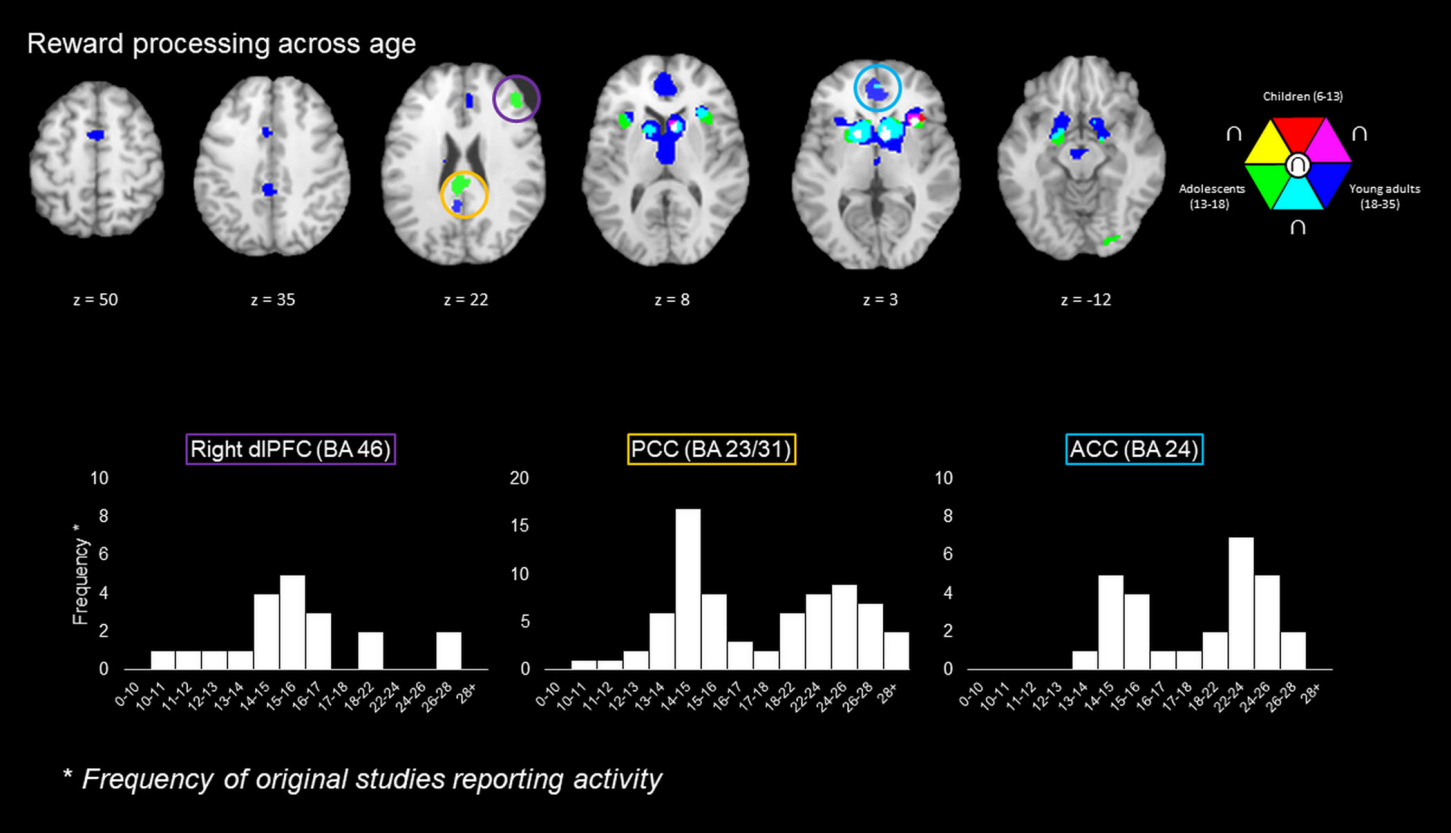
(Top) Concordant brain activity of reward processing across studies for each age group. Result of the children meta‐analysis are represented in red, adolescents are represented in green and young adults are represented in dark blue. Overlap of each age group are represented in yellow (children ∩ adolescents), turquoise (adolescents ∩ adults), magenta (children ∩ young adults) and white (all). (Bottom) Bar graphs representing the frequency of relevant clusters (right dorsolateral prefrontal cortex [dlPFC], posterior cingulate cortex [PCC] and anterior cingulate cortex [ACC]) across age

Tables 1, 2, 3 shows a complete list of concordant brain regions associated with reward processing with stereotaxic coordinates in Talairach space identified by all ALE meta‐analyses by age group, conjunction and contrast analyses, respectively. Significant results were separated by age group and illustrated on Figure 1. Supplementary analyses on reward outcomes for each age group was performed since enough studies were available for each age group. Additional meta‐analyses include reward anticipation for all age groups combined, and a meta‐analysis on reward processing for monetary incentive delay task only (Tables S4–S6; Figure S3). Note that the latter two meta‐analyses were performed by combining all age groups for the purpose of exploring brain maps associated with these events. Supplementary analyses revealed concordance patterns similar to the main meta‐analyses: with exception of the insula, which shows no significant concordances during reward anticipation tasks.

**TABLE 1:** Concordant brain regions related to reward processing

Cluster #	Volume mm^3^	ALE value	x	y	z	Brain region
Children						
1	1,672	0.025	12	4	−4	R globus pallidus
		0.018	8	10	6	R caudate body
2	1,320	0.023	34	20	2	R insula BA 13
		0.020	30	18	2	R claustrum
3	1,072	0.021	−14	2	−6	L globus pallidus
		0.018	−14	6	4	L putamen
		0.015	−8	12	−2	L caudate head
Adolescents						
1	6,632	0.063	−10	6	0	L caudate head
		0.032	−18	−6	−12	L amygdala
2	5,392	0.078	12	12	−2	R caudate head
		0.020	18	−8	−12	R amygdala
3	2,416	0.033	−2	−30	28	L posterior cingulate gyrus BA 23
		0.029	−2	−38	26	L posterior cingulate gyrus BA 31
		0.020	0	−22	32	L posterior cingulate gyrus BA 23
4	1,312	0.032	32	18	6	R insula BA 13
5	904	0.037	−32	14	10	L insula BA 13
6	848	0.033	0	46	−4	L anterior cingulate gyrus BA 32
7	784	0.025	44	32	20	R middle frontal gyrus BA 46
		0.023	44	38	22	R middle frontal gyrus BA 46
		0.019	40	28	12	R inferior frontal gyrus BA 46
8	680	0.025	22	−90	−10	R fusiform gyrus BA 18
		0.022	30	−84	−10	R inferior occipital gyrus BA 18
Adults						
1	25,688	0.133	10	8	−2	R caudate head
		0.120	−10	8	0	L caudate head
		0.056	30	20	2	R insula BA 13
		0.046	2	−14	8	R thalamus (dorsal medial)
		0.043	2	−6	8	R thalamus (ventral lateral)
		0.036	−28	18	6	L claustrum
		0.026	−10	−16	12	L thalamus (dorsal medial)
		0.025	−14	−16	18	L thalamus (ventral lateral)
2	6,040	0.053	2	40	10	R anterior cingulate gyrus BA 32
		0.026	4	30	28	R anterior cingulate gyrus BA 32
3	1808	0.039	2	0	48	R medial frontal gyrus BA 6
		0.029	−4	10	34	R anterior cingulate gyrus BA 24
4	1744	0.037	−6	−28	−4	L thalamus
		0.031	−4	−20	−10	L midbrain
		0.028	−16	−26	−4	L midbrain
5	1,504	0.044	−2	−36	32	L posterior cingulate gyrus BA 31
6	1,080	0.033	−4	−50	22	L posterior cingulate gyrus BA 23
						

*Note:* Talairach coordinates (x, y, z) of brain regions surviving a cluster‐level threshold of *p* < .05 and a cluster forming threshold of *p* < .01 for single studies.

Abbreviations: ALE, activation likelihood estimate; BA, Brodmann area; L, left; R, right.

**TABLE 2:** Conjunction of brain regions related to reward processing

Cluster #	Volume mm^3^	ALE value	x	y	z	Brain region
Conjunctions						
Adolescents‐AND‐children						
1	1,432	0.025	12	4	−4	R Globus pallidus
		0.018	8	10	6	R caudate body
2	1,064	0.021	−14	2	−6	L Globus pallidus
		0.018	−14	6	4	L putamen
		0.015	−8	12	−2	L caudate head
3	480	0.022	34	18	2	R insula BA 13
		0.020	30	18	2	R claustrum
Adults‐and‐adolescents						
1	5,328	0.063	−10	6	0	L caudate head
		0.028	−18	−4	−12	L amygdala
2	4,968	0.078	12	12	−2	R caudate head
		0.020	18	−8	−12	R amygdala
3	784	0.032	32	18	6	R insula BA 13
4	544	0.030	−2	−34	28	L posterior cingulate gyrus BA 23
5	520	0.030	0	46	−2	L anterior cingulate gyrus BA 32
6	176	0.028	−30	16	10	L insula BA 13
Adults‐and‐children						
1	1,672	0.025	12	4	−4	R Globus pallidus
		0.018	8	10	6	R caudate body
2	1,072	0.021	−14	2	−6	L Globus pallidus
		0.018	−14	6	4	L putamen
		0.015	−8	12	−2	L caudate head
3	1,048	0.023	34	20	2	R insula BA 13
		0.020	30	18	2	R claustrum

*Note:* Talairach coordinates (x, y, z) of brain regions surviving a cluster‐level threshold of *p* < .05 and a cluster forming threshold of *p* < .01 for single studies.

Abbreviations: ALE, activation likelihood estimate; BA, Brodmann area; L, left; R, right.

**TABLE 3:** Contrasts of brain regions related to reward processing

Cluster #	Volume mm^3^	ALE value	x	y	z	Brain region
Contrasts						
Adolescents > adults						
1	992	3.352	−4	−26	22	L posterior cingulate gyrus BA 23
		3.290	−4	−30	20	L posterior cingulate gyrus BA 23
		2.155	−0.5	34.5	21	L posterior cingulate gyrus BA 23
2	584	3.090	12	16	−1.3	R caudate body
3	464	3.238	−36	12	10	L insula BA 13
		3.194	−34	10	14	L insula BA 13
		3.061	−30	8	8	L claustrum
4	304	3.540	43	30	16	R middle frontal gyrus BA 46
		2.549	46	36	22	R middle frontal gyrus BA 46
5	208	2.770	36	14	8	R insula BA 13
Adults > children						
1	2,752	3.890	7.3	−12	6	R thalamus (dorsal medial)
		3.719	18	−6.7	0.7	R globus pallidus (medial)
		3.540	22	−4	0	R globus pallidus (lateral)
		2.911	13	−3	2	R midbrain
		2.862	18	12	2	R putamen
2	448	3.431	−6	−46	22	L posterior cingulate gyrus BA 30
		3.194	−4	−46	26	L posterior cingulate gyrus BA 31
		3.155	−2	−50	26	L posterior cingulate gyrus BA 31
		3.121	−6	−51	28	L posterior cingulate gyrus BA 31
3	304	2.820	−4	−42	30	L posterior cingulate gyrus BA 31
4	264	2.635	3	30	16	R anterior cingulate gyrus BA 24
Adolescents > children						
1	1,480	3.719	10	17	−2	R caudate head
		3.540	14	15	0	R caudate head
		3.431	18	14	0	R putamen
2	1,288	3.011	−4	−42	22	L posterior cingulate gyrus BA 23
		2.770	1	−35	26	R posterior cingulate gyrus BA 31
		2.737	−8	−32	22	L posterior cingulate gyrus BA 23
		2.678	−2	−36	22	L posterior cingulate gyrus BA 23
Adults > adolescents						
No suprathreshold clusters						
Children > adolescents						
No suprathreshold clusters						
Children > adults						
No suprathreshold clusters						

*Note:* Talairach coordinates (x, y, z) of brain regions surviving a cluster‐level threshold of *p* < .05 and a cluster forming threshold of *p* < .01 for single studies.

Abbreviations: ALE, activation likelihood estimate; BA, Brodmann area; L, left; R, right.

#### 3.1.1 Children

Concordant clusters for processing rewards in children are found in the basal ganglia, insula (Brodmann Area [BA] 13) and claustrum. Basal ganglia nuclei include the globus pallidus, caudate body, caudate head, and putamen. No suprathreshold clusters were observed in the ventromedial prefrontal cortex.

#### 3.1.2 Adolescents

The highest ALE scores for adolescents were observed in the caudate head in large clusters that also included the amygdalae. Other regions implicated in reward processing in adolescents were the posterior cingulate cortex (BA 23/31), insula cortex (BA 13), anterior cingulate cortex (BA 32), middle and inferior frontal gyri (BA 46), and fusiform/occipital cortex (BA 18).

#### 3.1.3 Adults

Concordant clusters for adults showed the highest ALE scores in the caudate head, insula (BA 13), and anterior cingulate gyrus (BA 32). Other regions implicated in reward processes in adults include the thalamus, claustrum, midbrain, medial frontal gyrus (BA6), anterior cingulate gyrus (BA 24), and posterior cingulate gyrus (BA 31/23).

#### 3.1.4 Adolescents versus children

Contrast and conjunction analyses between adolescents and children revealed greater concordance within the caudate head/putamen, and posterior cingulate gyri for adolescents compared to children. Conjunction analyses show common regions of concordance in the globus pallidus/caudate head, and insula/claustrum. No subrathreshold clusters were greater in children than adolescents.

#### 3.1.5 Adults versus children

Contrasts analysis revealed that adults engage the thalamus/globus pallidus/midbrain posterior cingulate and anterior cingulate cortices more extensively compared to children. The reverse contrast revealed no suprathreshold clusters. Conjunction analysis between adults and children revealed concordance in globus pallidus/caudate body, globus pallidus/putamen/caudate head and insula (BA 13)/claustrum.

#### 3.1.6 Adolescents versus adults

Contrast analysis revealed greater concordance within the posterior cingulate cortex (BA 23), caudate body, insula cortex/claustrum (BA 13), middle frontal cortex (BA 46) and insula cortex (BA 13) for adolescents compared to adults. Despite a larger sample size in the adult meta‐analysis, the reverse contrast revealed no suprathreshold clusters. Conjunction analysis resulted in common regions in the caudate head/amygdala, insula cortex (BA 13), and anterior/posterior cingulate cortices (BA 23/32). Since parts of the left caudate, left insula and left posterior cingulate cortices were included in both contrast and conjunction analyses, this suggests that both adults and adolescents recruit similar locations, yet more extensively for adolescents within the left caudate, insula cortex and posterior cingulate cortex.

#### 3.1.7 Post hoc analysis

**FIGURE 2:**
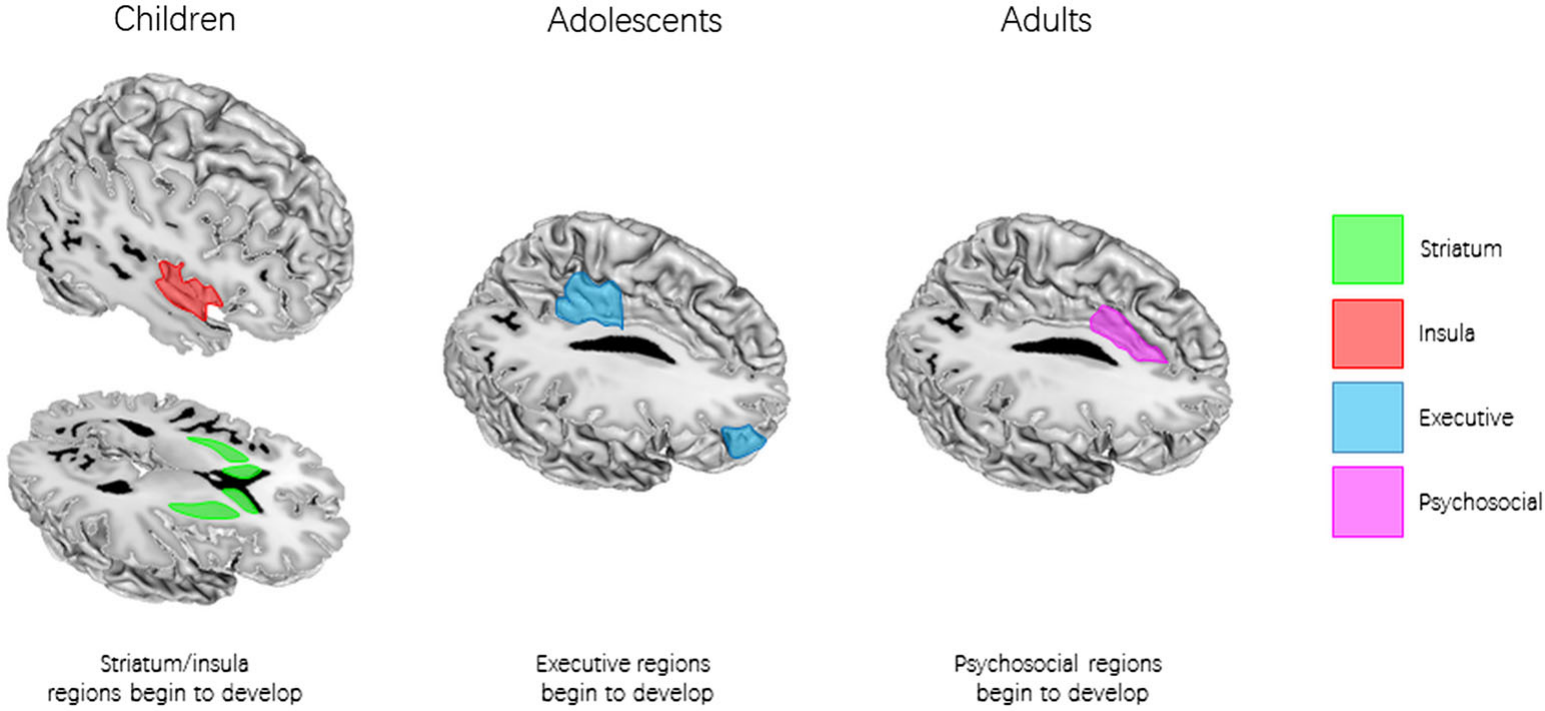
Diagram representing the proposed model. Striatum and insula are represented in green and red, respectively. These regions are present in all age groups yet emerge in early developmental stages. Executive and psychosocial regions consist of the prefrontal and cingulate regions and are represented in light blue. These areas emerge during adolescence and adulthood

To assess any systematic activity across different age groups we tested the frequency of foci reported with multiple bins associated with age for three key regions: the right dorsolateral prefrontal cortex, posterior cingulate cortex, and anterior cingulate cortex. We explored this relation by extracting foci from the raw data which fell within a 10 mm^3^ radius of the peak cluster from the main meta‐analysis. These values were then plotted in a histogram and viewed for changes across age (see bottom of Figure 1 for these histograms). These histograms revealed key developmental shifts in these regions, namely an abundance of articles increasing between 14 and 17 among all three regions, yet another increase in prevalent reports between 18 and 26 for the posterior and anterior cingulate clusters only. These findings may support the hypothesis that executive and psychosocial (e.g., emotional and social) abilities develop at different stages (Steinberg, 2007), implying that the right dorsolateral prefrontal cortex processes executive functions, and the cingulate cortex underlies psychosocial processing (also see Lieberman, Straccia, Meyer, Du, & Tan, 2019). This notion is best illustrated in a proposed biological model based on Steinberg's hypothesis (Steinberg, 2007; Figure 2). While executive regions develop during adolescence, the anterior cingulate specifically develops further during adulthood.

## 4 DISCUSSION

In a series of quantitative meta‐analyses we investigate concordance in brain responses to reward processing in children, adolescents, and adults. Specifically, we examine common and distinct executive and subcortical brain regions across different age groups. From these meta‐analyses we reveal that: (a) children show concordance in subcortical regions, yet lack implication of brain regions associated with the executive system; (b) adolescents recruit analogous subcortical regions as children yet they also engage the anterior and posterior cingulate cortices, amygdala and middle frontal gyrus (i.e., dorsolateral prefrontal cortex), and; (c) multiple regions (i.e., posterior cingulate gyrus, basal ganglia, insula, and middle frontal gyrus) in adolescence appear to be hyperactive when compared to adults.

In general, these findings support the notion that all age groups recruit the subcortical system, yet differences by age group rely on brain areas associated with the executive system. This is the first study that examined concordant brain areas among children, adolescents and adults, which allowed us to assess the pivotal moments of implication of certain executive regions (dorsolateral prefrontal cortex, anterior cingulate cortex and the posterior cingulate cortex) by plotting the frequency of these regions across age. Specifically, development of the executive component of reward processing seems to involve two dependent components maturing at different developmental stages: a logical‐reasoning component and a psychosocial/motivational component (Steinberg, 2007). Whereas adolescents are thought to attain adult‐like reasoning by age 15, psychosocial abilities are thought to follow a more protracted linear development (Luciana & Collins, 2012; Luciana, Wahlstrom, Porter, & Collins, 2012; Steinberg, 2007; Steinberg et al., 2009). This mechanism has been illustrated in Figure 2. Consequentially, an executive system that is still developing, along with psychosocial factors may be hindered in adolescents who often make risky decisions in social settings, and thus brain responses of adolescents may be associated with more salient experiences of reward anticipation and the reception of reward outcomes (Chein et al., 2011). Throughout the following, we discuss the brains regions involved in reward processing in the attempt to emphasize their functional role in children, adolescents, and adults.

### 4.1 Dorsal anterior and posterior cingulate: Adolescents and adults

Dorsal anterior and posterior parts of the cingulate cortex are implicated in reward processing for adolescents and adults. Specifically, comparing across age groups, we found no concordant cingulate clusters for children yet found concordant clusters in dorsal anterior and posterior cingulate gyri for adolescents and adults, thereby supporting the notion that the cingulate implication becomes more important during adolescence. When examining the histograms of the dorsal anterior and posterior cingulate cortices, coordinates in the dorsal anterior and posterior cingulate cortex were reported at higher rates by studies that examined age groups between 14 and 16 years, and between 22 and 26 years, as illustrated in the lower panel of Figure 1.

The anterior cingulate cortex is a functionally heterogeneous region that is anatomically connected to various anterior and posterior regions (Vogt, Finch, & Olson, 1992) including the prefrontal cortex (Barbas, 2015; Ray & Zald, 2012; Yeterian, Pandya, Tomaiuolo, & Petrides, 2012), but also subsections of the cingulate including the subgenuate, pregenuate, postgenuate, dorsal anterior cingulate areas (Mao et al., 2017; Palomero‐Gallagher et al., 2019; Stevens, Hurley, & Taber, 2011). The anterior cingulate cortex may be related to detection of prediction errors in monetary (Brown & Braver, 2005; Hauser et al., 2014; Holroyd & Coles, 2002; Garrison et al., 2013) and social contexts (Eisenberger & Lieberman, 2004; Lockwood et al., 2015; van der Molen, Dekkers, Westenberg, van der Veen, & van der Molen, 2017; Lockwood & Wittmann, 2018). Moreover, the anterior cingulate cortex may play a crucial role in motivated social cognition (Apps, Rushworth, & Chang, 2016; Eisenberger & Lieberman, 2004; Hughes & Beer, 2012; Park et al., 2016; van der Molen et al., 2017; Wittmann, Lockwood, & Rushworth, 2018) perhaps by estimating the motivation of others and updating this information based on erroneous predictions (Apps et al., 2016).

A specific methodological consideration is that adult task protocols may reflect higher demands compared with those used in younger children and should be regarded in the interpretation of the results. An alternative interpretation to the lack of cingulate activity in children may be that adolescents and adults may monitor performance and thereby experience error‐related processing differently than children, such that children rely on model‐free decision‐making processing each trial more independently (Decker et al., 2016). This would support the notion that children lack specific cognitive abilities that would allow one to regulate decision‐making (e.g., Arsalidou & Pascual‐Leone, 2016).

In general, the anterior and posterior regions of the cingulate cortex are associated with the detection and monitoring of change or unexpected stimuli (Pearson et al., 2009; Pearson et al., 2011; Apps et al., 2012). Within the context of reward, while the anterior cingulate cortex is involved in the experience of pleasure or happiness (Lindgren et al., 2012; Matsunaga et al., 2016; Rolls et al., 2003; Suardi, Sotgiu, Costa, Cauda, & Rusconi, 2016), and value‐guided decision‐making (Holroyd & Coles, 2002; Kolling et al., 2016; Shenhav, Cohen, & Botvinick, 2016), the posterior cingulate cortex involves the monitoring of action‐reward outcome associations (Hayden, Nair, McCoy, & Platt, 2008; Tabuchi et al., 2005). Together, anterior and posterior cingulate cortices have been associated with different aspects of motivation; the anterior cingulate processes motivational choices for complex cognitive tasks (i.e., decision‐making) while the posterior cingulate processes self‐referential motivational choices. Neurologically, the relative increase in cingulate foci reported in studies may be explained by cerebral developments at the onset of puberty such as pruning or redundant synaptic connectivity and myelination, which continue to develop into early adulthood (Giedd et al., 1999; Kelly et al., 2008; Rakic, Bourgeois, & Goldman‐Rakic, 1994).

### 4.2 Dorsolateral prefrontal cortex: Adolescents

Along with the anterior cingulate cortex, the dorsolateral prefrontal cortex is typically associated with higher order cognitive and executive control functions such as conflict/error detection (Koechlin, Ody, & Kouneiher, 2003; Botvinick, Cohen, & Carter, 2004), response inhibition (Aron et al., 2006; Hampshire, Chamberlain, Monti, Duncan, & Owen, 2010; Hampshire, Thompson, Duncan, & Owen, 2009; Hung, Gaillard, Yarmak, & Arsalidou, 2018), working memory (Owen, McMillan, Laird, & Bullmore, 2005; Rottschy et al., 2012; Yaple & Arsalidou, 2018), and negative priming (Frings, Schneider, & Fox, 2015; Yaple & Arsalidou, 2018). The dorsolateral prefrontal cortex is generally associated with integration of information (Krawczyk, 2002; Liu et al., 2011; Lorenz et al., 2014) and maintaining externally generated information (Christoff & Gabrielli, 2000; Christoff et al., 2009). Studies focusing on reward processing also show right dorsal lateral prefrontal cortex when receiving gains (van den Bos, Crone, & Güroğlu, 2012; Yaxley et al., 2011) as well as source estimation of electrophysiological oscillatory components (Haji Hosseini & Holroyd, 2015; Yaple et al., 2018). In the current meta‐analyses, dorsolateral prefrontal cortex (middle and inferior frontal gyri, BA 46) was concordant in the meta‐analysis with adolescents, confirming the hypothesis that changes in reward‐seeking behavior during adolescence may occur from an increased recruitment of the top‐down control component (Steinbeis et al., 2014).

According to theoretical predictions, mental‐attentional capacity, expressed by dorsolateral prefrontal cortex activity, continues to develop such that 13–14, and 15–16 years olds can hold on average 6 and 7 items in mind (Pascual‐Leone, 1970; Pascual‐Leone & Johnson, 2005). Thus, during adolescence more resources underlined by dorsolateral prefrontal cortex may come online in an effort to resolve problems associated with decision‐making. Notably, the previous meta‐analyses on adolescents (Silverman et al., 2015) did not report suprathreshold clusters in dorsolateral prefrontal cortex, likely because in their study selection a broader age range with children as young as 8 and 9 years was used. Our analyses that distinguishes between younger children and adolescence shows no suprathreshold concordance in the prefrontal cortex of younger children, consistent with past meta‐analyses that suggests reorganization of prefrontal function in younger age groups (Yaple & Arsalidou, 2018; Arsalidou et al., 2018). Consistently, a biological explanation could suggest an ongoing maturation of the prefrontal cortex during childhood and adolescents (Gogtay et al., 2004; Petanjek, Judaš, Kostović, & Uylings, 2007). For example, adolescent development undergoes dramatic dendritic and synaptic changes in the prefrontal cortex (Huttenlocher & Dabholkar, 1997; Koss, Belden, Hristov, & Juraska, 2014; Shapiro, Parsons, Koleske, & Gourley, 2017), which may in part account for the greater increase in likelihood of activation across studies.

### 4.3 Striatum: Children, adolescents, and adults

Subregions of the striatum such as the globus pallidus, caudate and putamen were concordant in the meta‐analyses of all age groups. Basal ganglia nuclei are associated with a variety of cognitive, emotional, and reward‐related processes (Arsalidou, Duerden, & Taylor, 2013; Arsalidou et al., in press for meta‐analyses). Reward‐related processes that implicate these regions include probabilistic feedback (Aron et al., 2004; Foerde, Knowlton, & Poldrack, 2006; Poldrack et al., 2001; Schwabe, Tegenthoff, Höffken, & Wolf, 2013; Schwabe & Wolf, 2012) and sequential decision‐making (Doll, Duncan, Simon, Shohamy, & Daw, 2015; Lee, Shimojo, & O'Doherty, 2014; Nebe et al., 2018). The caudate integrates valuation with action (Haber & Knutson, 2010), which accords with the current findings since all age groups activated this region across studies, and all participants across age groups were asked to make a cognitive valuation followed by a motor response. The dorsal striatum has been suggested to be involved in encoding of habitual learning (Patterson & Knowlton, 2018) and with learning new stimulus–reward contingencies (Knutson & Cooper, 2005; Rogers et al., 2004). Since dorsal parts of the basal ganglia have been implicated in processing rewards in children, adolescents, and adults we propose that these subcortical regions develop early with respect to cortical regions. This is consistent with the theory of constructive operators, which suggest that fundamental aspects of the Affective (A)‐operator, housed in the limbic system, are ontologically and phylogenetically the first to develop (e.g., Arsalidou & Pascual‐Leone, 2016; Pascual‐Leone & Johnson, 2005).

### 4.4 Amygdala: Adolescents, and adults

Large clusters that peaked over the caudate extended to the amygdala for both the adolescent and adult groups. We also find amygdala to be significantly concordant in the conjunction of these two groups. The amygdala is traditionally associated with emotional learning (Huff, Miller, Deisseroth, Moorman, & LaLumiere, 2013; Nieh, Kim, Namburi, & Tye, 2013) and processing of fear conditioning and anxiety (LaLumiere, 2014; LeDoux, 2000; Maren & Quirk, 2004; Nieh et al., 2013; Pape & Pare, 2010); however, it is also a key area of the mesolimbic dopamine reward system which projects to the nucleus accumbens during rewarding events (Nieh et al., 2013).

### 4.5 Insula and claustrum: Children, adolescents, and adults

Laterally adjacent to the dorsal striatum are the claustrum and insular cortex, which were also found to be concordant across studies in all three age groups. Along with the anterior cingulate cortex, the insula is another region that activates to an array of cognitive, emotional and interoceptive events, to which some have suggested that these regions are key nodes in a salience network associated with responding to stimuli deserving of attention (Calder, Keane, Manes, Antoun, & Young, 2000; Calder, Lawrence, & Young, 2001; Menon & Uddin, 2010). In a coordinate‐based meta‐analysis it was revealed that the insula assumes multiple functions, anatomically portrayed as a topographic map (Kurth, Zilles, Fox, Laird, & Eickhoff, 2010). Specifically, the anterior‐dorsal part of the insula was found to be associated with executive/cognitive functions, while the anterior‐ventral part corresponds with social–emotional functions such as emotional processing and empathy. The idea that the insula may be related to motivated cognitive behavior has been proposed in earlier developmental studies of working memory (Yaple & Arsalidou, 2018) and mathematical cognition (Arsalidou et al., 2018).

Some reward‐related studies suggest that the insula is primarily involved in the processing of negative events (Phillips et al., 1998; Morris, Scott, & Dolan, 1999; Davis et al., 2010). Others later have shown that the insula involves both gains and losses (Camara, Rodriguez‐Fornells, & Münte, 2009; Choi, Padmala, Spechler, & Pessoa, 2014). Systematic reviews on reward processing have suggested that the insula responds to expectation of rewards (Knutson & Bossaerts, 2007; Knutson & Greer, 2008; Liu et al., 2011; Moreira, Pinto, Almeida, Barros, & Barbosa, 2016), yet other studies have found that the insula responds to reward anticipation as well as reward delivery (Boecker et al., 2014; Liu et al., 2011; Padmala & Pessoa, 2011; Samanez‐Larkin et al., 2007). To address this inference, we emphasize the results of the supplementary meta‐analyses on *reward anticipation* and *reward outcome* across age groups, revealing concordant activity within the insula for reward anticipation, but not reward outcome. This supports the notion that insula may not necessarily be functionally associated with observing reward outcomes (See Table S4; Figure S3).

Interestingly, previous meta‐analyses on cognitive abilities in children revealed concordant right‐lateralized insula cortex activity, suggesting that right insula cortex activation is essential for problem solving (Yaple & Arsalidou, 2018; Arsalidou et al., 2018). In the current research, we found this region to be highly significant, especially within the meta‐analysis on reward anticipation (See Table S5; Figure S3). Because the insulae is implicated in different constructs related to rewards as well as other qualitative different tasks (e.g., n‐back; Yaple & Arsalidou, 2018), we speculate a generic role of the insula; involving multiple functions including the processing of rewards. Critically our results developmentally confirm the implication of the insular cortex in reward processes as it appears in conjunction results for all age groups. Perhaps the insulae may serve as moderator between affective and cognitive processes as they relate to motivation to avoid aversive stimuli and sustaining performance in a task, as suggested by developmental theory (e.g., Arsalidou & Pascual‐Leone, 2016; Arsalidou et al., 2018; Yaple & Arsalidou, 2018).

### 4.6 Limitations

Our meta‐analyses evaluated coordinates from fMRI studies that examined reward processes in children, adolescents, and adults. To achieve sufficient power for the analyses we cotableh study heterogeneity. To this regard, we omitted contrasts that included monetary losses to specifically focus on reward processing. In addition, we separately performed secondary analyses on reward outcomes, reward anticipation and a task‐relevant dataset across all age groups (see Supplementary Materials section). We had initially considered performing separate meta‐analyses on losses, risk taking and delay discounting; yet the number or reported articles were insufficient. When further data becomes available, future meta‐analyses can address specific questions related to these processes across age.

Further, the number of studies considered for each age group was different with the least number of studies in the children group; albeit all age‐related analyses reported in the main text adhere to minimum experiment requirements for sufficient statistical power (Eickhoff et al., 2017). These are a main disadvantage of performing meta‐analyses across age groups, however, as this is the nature of tasks variability in reward processes in the literature, it is the state of the art. Optimally, future developmental studies should consider parametric tasks with a common goal but variable levels of difficulty to ensure that individuals with variable performance levels can complete the task (e.g., Arsalidou & Im‐Bolter, 2017). Finally, many studies were not included in our meta‐analyses due to the relatively wide in range in age. We encourage future research in this field to focus on discrete or narrower age ranges, as opposed to studies using a wider age range to allow for improved option for determining the relative shifts in brain activity throughout development.

## 5 CONCLUSIONS

In these large‐scale meta‐analyses with a total of 554 children, 1,059 adolescents, and 1,831 adults, we showed that all age groups yield consistent activity in the striatum and the insula. Children lack concordant activation of regions implicated in associative “higher‐order” regions. Across studies, adolescents engage the right dorsolateral prefrontal cortex, a key region involved in executive control, whereas adults show concordance in anterior cingulate cortex but no concordant activity within the dorsolateral prefrontal cortex. Our findings suggest that these executive regions undergo dramatic changes across adolescence through to adulthood. These findings coincide with the notion that these executive regions may develop twofold: distinguished by dorsolateral prefrontal cortex concordance in adolescents representing the development of executive control processing at around 15 years of age, and anterior cingulate cortex concordance signifying later development of psychosocial abilities in early adulthood.

## Supporting information


**Figure S1** PRISMA flowchart for eligibility of articles for adult meta‐analyses.

**Figure S2** PRISMA flowchart for eligibility of articles for meta‐analyses in children and adolescents groups; ^a^ = three studies were included in both groups (Cohen et al., 2010; Van Leijenhorst et al., 2010; Paulsen, Carter, Platt, Huettel, & Brannon, 2012).

**Figure S3** (Top) Concordant brain activity of reward outcomes across studies for each age group. Result of the children meta‐analysis are represented in red, adolescents are represented in green and young adults are represented in dark blue. Overlap of each age group are represented in yellow (children ∩ adolescents), turquoise (adolescents ∩ adults), magenta (children ∩ young adults) and white (all). (Middle) Concordant brain activity of reward anticipation across all age groups (i.e., children, adolescents and adults were considered together). (Bottom) Concordant brain activity of the Monetary Incentive Delay (MID) task across all age groups (i.e., children, adolescents and adults were considered together).

**Table S1** **.** Information on source datasets included in the meta‐analysis for children.
**Table S2.** Information on source datasets included in the meta‐analysis for adolescents.
**Table S3**. Information on source datasets included in the meta‐analysis for early adults
**Table S4.** Concordant brain regions related to reward outcomes for each age group
**Table S5.** Concordant brain regions related to reward anticipation.
**Table S6.** Concordant brain regions related to monetary incentive delay (MID) task.

## Data Availability

Development of reward processing: Over‐arching brain clusters in children, adolescents, and adults. The data that support the findings of this study are openly available in OSF at DOI 10.17605/OSF.IO/5XUQW Data sharing is not applicable to this article as no new data were created or analyzed in this study.
