# Supplementary figures and images for: Spatial migration of human reward processing with functional development: Evidence from quantitative meta‐analyses
Source: Hum Brain Mapp. 2020 Jul 7;41(14):3993–4009. doi: 10.1002/hbm.25103 (PMC7469823; doi:10.1002/hbm.25103)

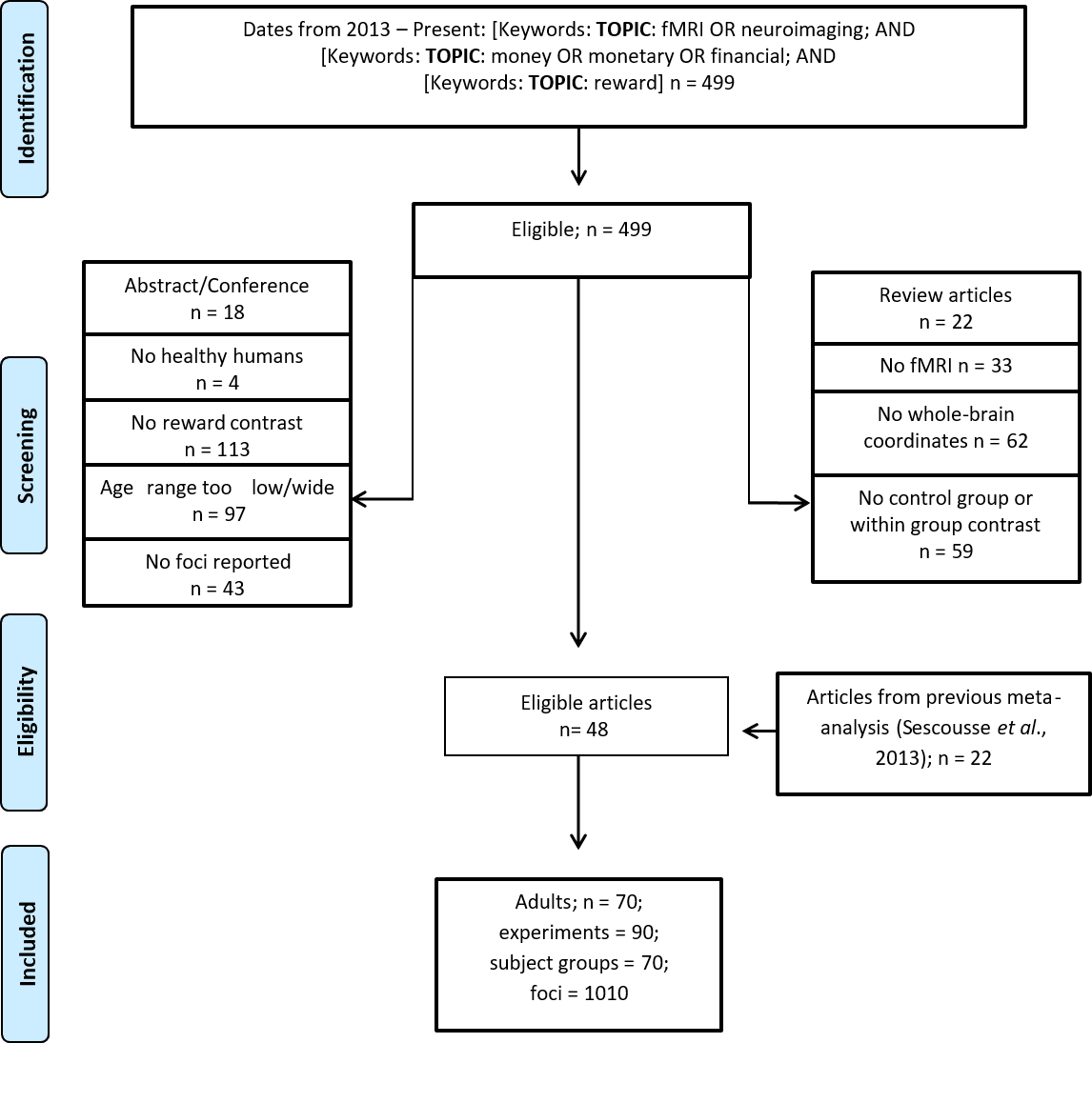

Supplement: Supplementary file 1 — Figure S1 PRISMA flowchart for eligibility of articles for adult meta‐analyses. [file HBM-41-3993-s001.tif]

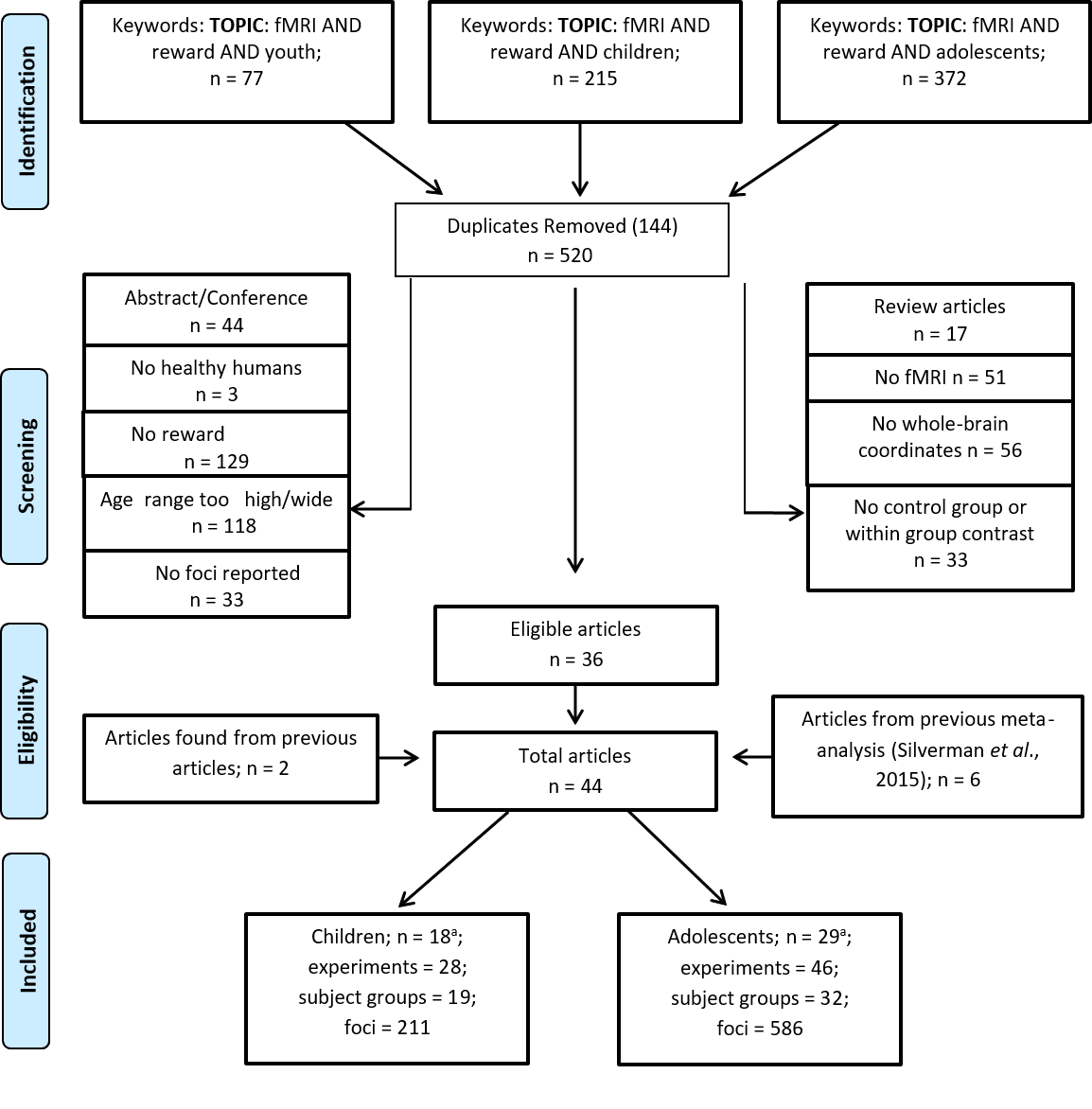

Supplement: Supplementary file 2 — Figure S2 PRISMA flowchart for eligibility of articles for meta‐analyses in children and adolescents groups; a = three studies were included in both groups (Cohen et al., 2010; Van Leijenhorst et al., 2010; Paulsen, Carter, Platt, Huettel, & Brannon, 2012). [file HBM-41-3993-s002.tif]

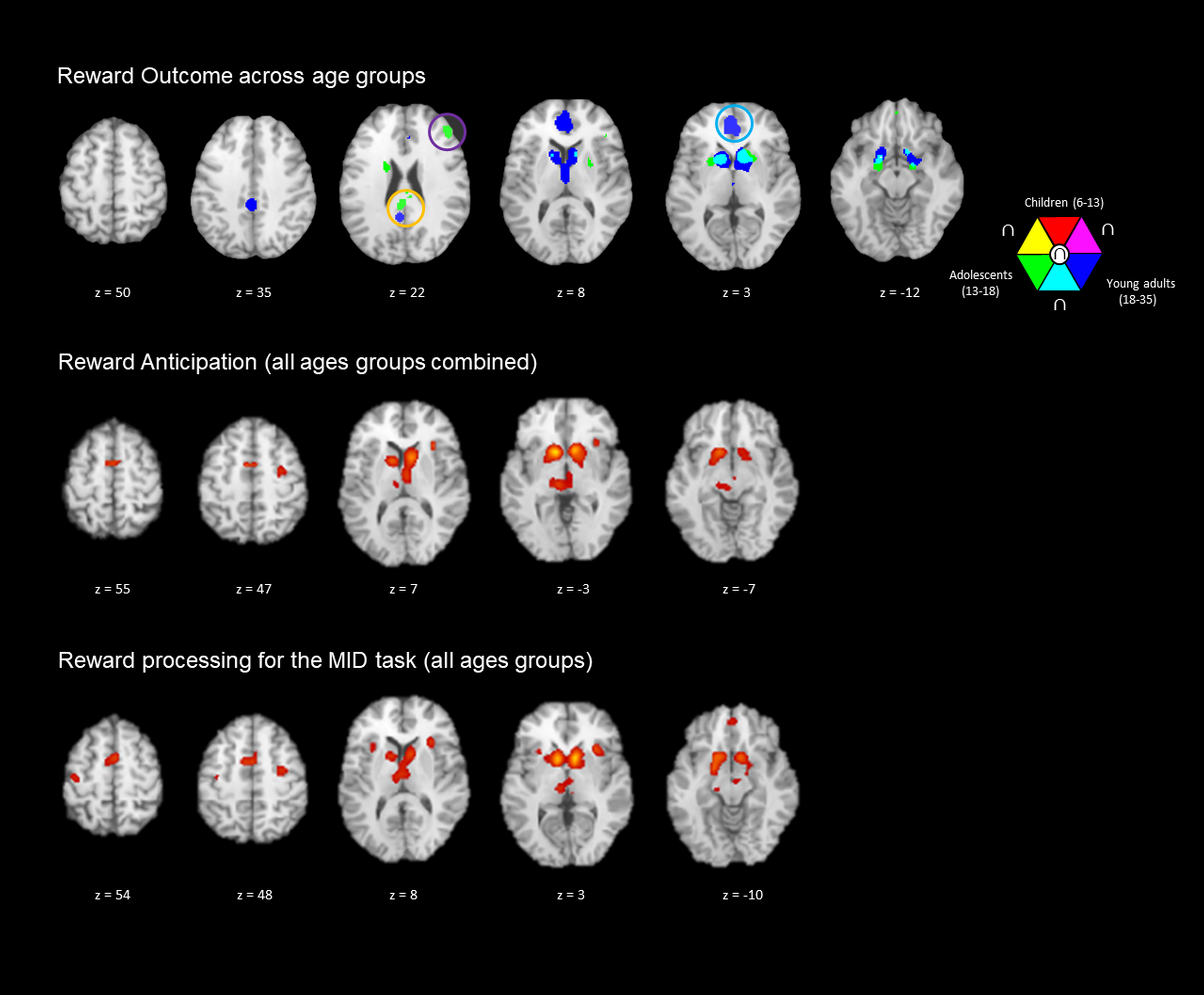

Supplement: Supplementary file 3 — Figure S3 (Top) Concordant brain activity of reward outcomes across studies for each age group. Result of the children meta‐analysis are represented in red, adolescents are represented in green and young adults are represented in dark blue. Overlap of each age group are represented in yellow (children ∩ adolescents), turquoise (adolescents ∩ adults), magenta (children ∩ young adults) and white (all). (Middle) Concordant brain activity of reward anticipation across all age groups (i.e., children, adolescents and adults were considered together). (Bottom) Concordant brain activity of the Monetary Incentive Delay (MID) task across all age groups (i.e., children, adolescents and adults were considered together). [file HBM-41-3993-s003.tiff]
