# Supplementary material for: Spatial migration of human reward processing with functional development: Evidence from quantitative meta‐analyses
Source: Hum Brain Mapp. 2020 Jul 7;41(14):3993–4009. doi: 10.1002/hbm.25103 (PMC7469823; doi:10.1002/hbm.25103)
Supplement: Supplementary file 4 — Table S1 . Information on source datasets included in the meta‐analysis for children. Table S2. Information on source datasets included in the meta‐analysis for adolescents. Table S3. Information on source datasets included in the meta‐analysis for early adults Table S4. Concordant brain regions related to reward outcomes for each age group Table S5. Concordant brain regions related to reward anticipation. Table S6. Concordant brain regions related to monetary incentive delay (MID) task. [file HBM-41-3993-s004.docx]

**Supplementary Materials**:

SM Table 1. Information on source datasets included in the meta-analysis for children

| Article | | n | Male | | Mean (SD); range | | Foci | Task | Contrast type | RT (ms); | Acc % | | |  |  |  |  |  |  |
| --- | --- | --- | --- | --- | --- | --- | --- | --- | --- | --- | --- | --- | --- | --- | --- | --- | --- | --- | --- |
| Cohen *et al.*, 2010 ^c^ | | 18 | 9 | | 10.8; 8-12 | | 1 | PLT | Reward Outcomes | 901.6 | 63.4 | | |  |  |  |  |  |  |
| Crowley *et al.*, 2017 | | 58 | 27 | | 10.6 (0.8); 9-11 | | 16 | Colorado Balloon | Reward Risk | 292 | 92.9 | | |  |  |  |  |  |  |
| de Macks *et al.*, 2016 ^a^ | | 78 | 0 | | 11.87 (0.81); 11-13 | | 6 | Jackpot | Reward Outcomes | ~1010 | ~50 | | |  |  |  |  |  |  |
|  | |  |  | |  | | 7 | Jackpot | Reward Outcomes | ~990 | ~42 | | |  |  |  |  |  |  |
| Edmiston *et al.*, 2015 ^a^ | | 16 | 13 | | 10.13 (1.52); 8-12 | | 1 | Prisoner’s dilemma | Reward Outcomes | 1016 | NA | | |  |  |  |  |  |  |
|  | |  |  | |  | | 11 | Prisoner’s dilemma | Reward Outcomes | 1005 | NA | | |  |  |  |  |  |  |
|  | |  |  | |  | | 4 | Prisoner’s dilemma | Reward Outcomes | 1067 | NA | | |  |  |  |  |  |  |
| Forbes *et al.*, 2010 ^ab^ | | 26 | 8 | | 11.42 (0.58); 11-13 | | 17 | Card Guessing | Reward Anticipation |  |  | | |  |  |  |  |  |  |
|  | |  |  | |  | | 13 | Card Guessing | Reward Outcomes | 1105.23 | NA | | |  |  |  |  |  |  |
| Forbes *et al.*, 2010 ^ab^ | | 51 | 30 | | 12.2 (0.69); 11-13 | | 13 | Card Guessing | Reward Anticipation |  |  | | |  |  |  |  |  |  |
|  | |  |  | |  | | 13 | Card Guessing | Reward Outcomes | 1083.96 | NA | | |  |  |  |  |  |  |
| Helfinstein *et al.*, 2012 | | 50 | 25 | | 11 (2.14); 8-12 | | 3 | MID | Reward Anticipation | 510.8 | 57.5 | | |  |  |  |  |  |  |
| Ivanov *et al*., 2012 | | 20 | 18 | | 10.53±1.44; 8-12 | | 2 | ACR | Reward Anticipation |  |  | | |  |  |  |  |  |  |
|  | |  |  | |  | | 2 | ACR | Reward Outcomes | 493 | 88 | | |  |  |  |  |  |  |
| Joseph *et al.*, 2016 | | 24 | 10 | | ~12.5; 11-14 | | 9 | MID | Risk-sensitive | NA | 61.9 | | |  |  |  |  |  |  |
| Kappel *et al.*, 2013 | | 10 | 8 | | 11 (0.4); 8-12 | | 13 | MID | Reward Anticipation | 323.18 | 52.49 | | |  |  |  |  |  |  |
| Lahat *et al*., 2018 | | 40 | 17 | | 10.48±0.42 | | 3 | MID | Reward Outcomes | 454.53 | 58.05 | | |  |  |  |  |  |  |
| Paulsen *et al.*, 2012 ^bc^ | | 17 | 9 | | 6.9; 5.9-8 | | 10 | Monetary Gambling | Reward Outcomes | 660 | NA | | |  |  |  |  |  |  |
| Schlund *et al.*, 2011 | | 15 | 8 | | 11.1 (1.6); 9-13 | | 4 | Avoidance paradigm | Approach | NA | NA | | |  |  |  |  |  |  |
| Scott-van Zeeland *et al.*, 2010 ^a^ | | 16 | 16 | | 12.3 (1.76) | | 3 | PLT | Reward Outcomes | NA | 74.39 | | |  |  |  |  |  |  |
|  | |  |  | |  | | 11 | PLT | Reward Outcomes |  |  | | |  |  |  |  |  |  |
| Steinbeis *et al.*, 2014 | | 20 | 10 | | 9.7; 6.6-12.7 | | 3 | Delay discounting | Decision phase | NA | NA | | |  |  |  |  |  |  |
| Thomason & Marusak, 2017 | | 20 | 7 | | ~12.25 (2); 9-12 | | 10 | Reward | Reward Outcomes | 859.17 | 97.47 | | |  |  |  |  |  |  |
| van Leijenhorst *et al.*, 2006 ^a^ | | 12 | 5 | | 11.3 (0.9); 9-12 | | 8 | Cake gambling | Reward Risk | NA | 91 | | |  |  |  |  |  |  |
|  | |  |  | |  | | 1 | Cake gambling | Reward Risk |  |  | | |  |  |  |  |  |  |
|  | |  |  | |  | | 5 | Cake gambling | Reward Outcomes |  |  | | |  |  |  |  |  |  |
| van Leijenhorst *et al.*, 2010b ^abc^ | | 17 | 9 | | 11.6 (0.8); 10-12 | | 9 | Slot machine | Reward Anticipation | NA | NA | | |  |  |  |  |  |  |
|  | |  |  | |  | | 10 | Slot machine | Reward Outcomes |  |  | | |  |  |  |  |  |  |
| Wiggins *et al.*, 2017 | | 46 | 28 | | 7.54; 6-10 | | 3 | MID | Reward Outcomes | 340 | 70 | | |  |  |  |  |  |  |
|  |  | | |  | |  | | | | |  |  |  | |  |  |  |  |  |

Note: n = sample size; R = Right handed; SD = Standard deviation; NA = not available; ^a^ = article includes more than one contrast; ^b^ = article included from previous meta-analysis (Silverman et al., 2015); ^c^ = article included in adolescents/early adults search; PLT = Probabilistic learning task; MID = monetary incentive delay task

SM Table 2. Information on source datasets included in the meta-analysis for adolescents

| Article | n | Male | Mean (SD); range | | Foci | Task | | | Contrast type | | RT (ms) | | Acc (%) | |  |
| --- | --- | --- | --- | --- | --- | --- | --- | --- | --- | --- | --- | --- | --- | --- | --- |
| Banich *et al.*, 2013 | 29 | 14 | 16.11; 14-19 | | 16 | Delay discounting | | | Reward Risk | | 1700 | | NA | |  |
| Barkley-Levenson *et al.*, 2013 ^a^ | 19 | 9 | 15.5 (1.3); 13-17 | | 12 | Monetary Gambling | | | Reward Risk | | 1460 | | NA | |  |
|  |  |  |  | | 8 | Monetary Gambling | | | Reward Risk | |  | |  | |  |
| Bjork *et al.*, 2004 ^ac^ | 12 | 6 | 14.75 (1.9); 12-17 | | 8 | MID | | | Reward Outcomes | | NA | | NA | |  |
|  |  |  |  | | 17 | MID | | | Reward Outcomes | |  | |  | |  |
| Bjork *et al.*, 2008 ^ab^ | 13 | 8 | 13.9 (0.4); 12-16 | | 1 | MID | | | Reward Anticipation | |  | |  | |  |
|  |  |  |  | | 13 | MID | | | Reward Outcomes | | NA | | 69.6 | |  |
| Bjork *et al.*, 2008 ^ab^ | 13 | 8 | 13.8 (0.4); 12-16 | | 4 | MID | | | Reward Anticipation | |  | |  | |  |
|  |  |  |  | | 13 | MID | | | Reward Outcomes | | NA | | 69.6 | |  |
| Bjork *et al.*, 2010a ^a^ | 12 | 9 | 15.3 (1.4); 13-17 | | 7 | MID | | | Reward Anticipation | |  | |  | |  |
|  |  |  |  | | 6 | MID | | | Reward Outcomes | | 202 | | NA | |  |
| Bjork *et al.*, 2010b ^ac^ | 24 | 12 | 14.8 (1.8); 12-17 | | 13 | MID | | | Reward Anticipation | |  | |  | |  |
|  |  |  |  | | 9 | MID | | | Reward Outcomes | | ~250 | | ~75 | |  |
| Cohen *et al.*, 2010 ^bc^ | 16 | 10 | 15.8; 14-19 | | 1 | PLT | | | Reward Outcomes | | 908.9 | | 74.1 | |  |
| Crowley *et al.*, 2010 ^a^ | 20 | 20 | 16.5 (1); 14-18 | | 10 | Colorado Balloon | | | Decision phase | | 257 | | NA | |  |
|  |  |  |  | | 30 | Colorado Balloon | | | Reward Outcomes | |  | |  | |  |
| Cservenka *et al.*, 2012 ^b^ | 13 | 8 | 14.24 (0.75); 13-15 | | 4 | Wheel of fortune | | | Reward Risk | | NA | | NA | |  |
| Cservenka et al., 2012 ^b^ | 18 | 12 | 14.18 (0.7); 13-15 | | 6 | Wheel of fortune | | | Reward Risk | | NA | | NA | |  |
| Delgado-Rico *et al.*, 2013 | 13 | 5 | 13.69 (1.18); 12-17 | | 1 | Risky-gains | | | Reward Risk | | NA | | NA | |  |
| Gleich *et al.*, 2015 | 28 | 15 | 14.36 (0.62); 13-16 | | 10 | Slot machine | | | Reward Outcomes | | NA | | NA | |  |
| Helfinstein *et al.*, 2011 | 32 | 17 | 16.2; 14-18 | | 12 | Reward | | | Reward Outcomes | | NA | | NA | |  |
| Kim *et al.*, 2014 | 15 | 15 | 13.87 (0.83); 13-15 | | 2 | Reward | | | Reward Outcomes | | 165.49 | | 99 | |  |
| Kohls *et al.*, 2014 ^a^ | 17 | 17 | 13.9 (3) | | 10 | Incentive go/no-go | | | Reward Outcomes | | 231.4 | | 92.5 | |  |
|  |  |  |  | | 5 | Incentive go/no-go | | | Reward Outcomes | |  | |  | |  |
|  |  |  |  | | 5 | Incentive go/no-go | | | Reward Outcomes | |  | |  | |  |
| Navas *et al.*, 2018 ^a^ | 68 | 34 | 16.56 (1.35); 14-18 | | 25 | MID | | | Reward Anticipation | | 227 | | 0.58 | |  |
|  |  |  |  | | 21 | MID | | | Reward Outcomes | |  | |  | |  |
| Paloyelis *et al.*, 2012 | 30 | 30 | 15.42 (1.4) | | 9 | Incidental learning | | | Reward Anticipation | | 595.01 | | 78.48 | |  |
| Paulsen *et al.*, 2012 ^bc^ | 17 | 9 | 14.8; 14.2-15.9 | | 18 | Risky decision | | | Reward Outcomes | | 370 | | NA | |  |
| Peters *et al.*, 2011 | 86 | 48 | 14 (one age group) | | 28 | MID | | | Reward Anticipation | | 226.3 | | 70.09 | |  |
| Ripke *et al.*, 2012 ^a^ | 235 | 122 | 14.6 (0.3); 13-15.5 | | 20 | Delay discounting | | | Reward Outcomes | | NA | | NA | |  |
|  |  |  |  | | 21 | Delay discounting | | | Reward Risk | |  | |  | |  |
| Scheres *et al.*, 2007 | 11 | 8 | 13.9 (1.4); 12-17 | | 19 | Performance test | | | Reward Outcomes | | NA | | 58 | |  |
| Schneider *et al.*, 2012 ^a^ | 63 | 37 | 14 (one age group) | | 20 | MID | | | Reward Anticipation | | NA | | NA | |  |
|  |  |  |  | | 28 | MID | | | Reward Outcomes | |  | |  | |  |
| Schwenck *et al.*, 2017 | 24 | 24 | 14.34 (1.93); 11-17 | | 2 | Ultimatum-game | | | Reward Outcomes | | NA | | NA | |  |
| Telzer *et al.*, 2013 | 41 | 21 | 15.23; 14-16.5 | | 13 | Balloon analog risk | | | Reward Risk | | NA | | NA | |  |
| Vaidya *et al.*, 2013 ^c^ | 18 | 9 | 13.39 (0.92); 12-15 | | 13 | MID | | | Reward Outcomes | | 197 | | 67 | |  |
| van den Bos *et al.*, 2012 | 45 | 23 | 14.39; 13-16 | | 5 | Probabilistic learning | | | Reward Outcomes | | ~789 | | ~65 | |  |
| van Duijvenvoorde *et al.*, 2014 | 31 | 13 | 15.3 (2.1); 12-19 | | 9 | Jackpot | | | Reward Outcomes | | NA | | NA | |  |
| van Leijenhorst *et al.*, 2010a ^bc^ | 15 | 7 | 13.4 (0.8); 12-14 | | 23 | Cake gambling | | | Reward Outcomes | | ~1000 | | 65 | |  |
| van Leijenhorst *et al.*, 2010a ^abc^ | 15 | 8 | 17.1 (0.7); 16-17 | | 1 | Cake gambling | | | Reward Risk | | ~1200 | | 65 | |  |
|  |  |  |  | | 28 | Cake gambling | | | Reward Outcomes | |  | |  | |  |
| van Leijenhorst *et al.*, 2010b ^bc^ | 18 | 8 | 15 (0.7); 14-15 | | 11 | Slot machine | | | Reward Anticipation | | NA | | NA | |  |
|  |  |  |  | | 24 | Slot machine | | | Reward Outcomes | |  | |  | |  |
| Yaxley *et al.*, 2011 | 31 | 10 | 15.5 (1.5); 12.3-17 | | 25 | Reward uncertainty | | | Reward Outcomes | | 453 | | NA | |  |
|  |  |  |  |  | | |  |  | |  | |  | |  | |

Note: n = sample size; R = Right handed; SD = Standard deviation; NA = not available; ^a^ = article includes more than one contrast; ^b^ = article included from previous meta-analysis (Silverman et al., 2015); ^c^ = article included in children/early adults search; ; PLT = Probabilistic learning task; MID = monetary incentive delay task

SM Table 3. Information on source datasets included in the meta-analysis for early adults

| Article | n | Male | Mean (SD); range | Foci | Task | | Contrast type | | | | RT (ms) | | | | | Acc (%) |  |
| --- | --- | --- | --- | --- | --- | --- | --- | --- | --- | --- | --- | --- | --- | --- | --- | --- | --- |
| Albrecht *et al*., 2014 | 64 | 26 | 24.16: 18-34 | 2 | Picture-money | | Reward Outcomes | | | | NA | | | | | ~25 |  |
| Apaydin *et al*., 2018 | 18 | 7 | 25.8(5.8): 18-45 | 12 | Time estimation | | Reward Outcomes | | | | ~420 | | | | | ~76 |  |
| Barman *et al.*, 2015 | 63 | 32 | 24.55; 20.4-36.6 | 5 | MID | | Reward Anticipation | | | | 438 | | | | | 75.7 |  |
| Behan *et al*., 2015 | 20 | 9 | 23.05; 18-35 | 15 | MID | | Reward Anticipation | | | | ~280 | | | | | ~80 |  |
| Bijleveld *et al*., 2014 | 23 | 14 | 23.8(2.2) | 11 | Subliminal reward | | Reward Outcomes | | | | ~86 | | | | | ~863 |  |
| Bischoff-Grethe *et al*., 2015 | 20 | 8 | 27.7(8.53); 18-42 | 35 | Reward expectancy | | Reward Outcomes | | | | ~360 | | | | | NA |  |
| Bjork *et al*., 2004 ^abc^ | 12 | 6 | 23.8(2); 21-28 | 13 | MID | | Reward Anticipation | | | | NA | | | | | 69.9 |  |
|  |  |  |  | 16 | MID | | Reward Outcomes | | | |  | | | | |  |  |
| Bjork *et al*., 2010b ^ab^ | 24 | 12 | 29.3(5.7); 22-42 | 10 | MID | | Reward Anticipation | | | |  | | | | |  |  |
|  |  |  |  | 7 | MID | | Reward Outcomes | | | | ~250 | | | | | ~75 |  |
| Boecker *et al.*, 2014 | 162 | 94 | 24.4 | 5 | MID | | Reward Outcomes | | | | 195.81 | | | | | NA |  |
| Bothe *et al.*, 2013 | 43 | 43 | 24.77; 20-32 | 6 | MID | | Reward Outcomes | | | | NA | | | | | NA |  |
| Brevers *et al.*, 2016a | 15 | 6 | 22.07(1.67) | 4 | Iowa Gambling | | Reward Risk | | | | NA | | | | | NA |  |
| Camara *et al*., 2009 ^ab^ | 17 | 7 | 21.6(2.6) | 16 | Monetary Gambling | | Reward Outcomes | | | | NA | | | | | NA |  |
|  |  |  |  | 18 | Monetary Gambling | | Reward Outcomes | | | |  | | | | |  |  |
| Canessa *et al.*, 2013 | 56 | 27 | 24.84; 19-38 | 15 | Monetary Gambling | | Reward Anticipation | | | | NA | | | | | NA |  |
| Causse *et al.*, 2013 ^a^ | 15 | NA | 25.4(2.45) | 9 | Decision making | | Reward Risk | | | | 1223 | | | | | NA |  |
|  |  |  |  | 6 | Decision making | | Reward Outcomes | | | |  | | | | |  |  |
| Choi *et al.*, 2014 | 20 | 8 | 27.92(4.61) | 33 | MID | | Reward Anticipation | | | | 395 | | | | | NA |  |
| Clark *et al*., 2009 ^b^ | 15 | 9 | 26(7.5) | 18 | Slot machine | | Reward Outcomes | | | | NA | | | | | NA |  |
| Costumero *et al*., 2016 | 45 | NA | 26.44(5.39); 19-39 | 8 | Card Guessing | | Reward Outcomes | | | | NA | | | | | NA |  |
| Cox *et al*., 2005 ^b^ | 22 | 12 | 18-30 | 28 | Card Guessing | | Reward Outcomes | | | | 814 | | | | | NA |  |
| Dong *et al*., 2014 ^a^ | 31 | 31 | 22.8(3.4) | 4 | Card Guessing | | Reward Outcomes | | | | 366.98 | | | | | NA |  |
|  |  |  |  | 3 | Card Guessing | | Decision phase | | | |  | | | | |  |  |
|  |  |  |  | 2 | Card Guessing | | Reward Outcomes | | | |  | | | | |  |  |
| Elliott *et al*., 2003 ^ab^ | 12 | 6 | 23.6 | 12 | Target detection | | Reward Outcomes | | | | NA | | | | | NA |  |
|  |  |  |  | 10 | Target detection | | Reward Outcomes | | | |  | | | | |  |  |
| Ernst *et al*., 2005 ^ab^ | 14 | 8 | 26.7(5); 20-40 | 10 | Wheel of fortune | | Reward Outcomes | | | | NA | | | | | NA |  |
|  |  |  |  | 4 | Wheel of fortune | | Reward Outcomes | | | |  | | | | |  |  |
| Ferdinand & Opitz, 2014 | 17 | 10 | 23.4 (19-31) | 2 | Time estimation | | Reward Outcomes | | | | NA | | | | | NA |  |
| Fujiwara *et al*., 2009 ^b^ | 17 | 12 | 20-29 | 18 | Monetary Gambling | | Reward Outcomes | | | | ~1810 | | | | | NA |  |
| Funayama *et al*., 2014 | 20 | 12 | 29.9 | 17 | MID | | Reward Anticipation | | | | 224.22 | | | | | NA |  |
| Goerlich *et al*., 2016 | 45 | 45 | 24.1(3.2) | 4 | MID | | Reward Anticipation | | | | 242.4 | | | | | NA |  |
| Gossen *et al*., 2014 | 35 | 35 | 24.08 | 3 | MID | | Reward Anticipation | | | | 238.65 | | | | | 61.65 |  |
| Hardin *et al*., 2009 ^b^ | 18 | NA | 29 (4.8) | 7 | Wheel of fortune | | Reward Outcomes | | | | NA | | | | | NA |  |
| Hu *et al*., 2015 | 61 | 24 | 29.8(10) | 14 | Risk-taking | | Reward Risk | | | | 584 | | | | | 52.3 |  |
| Izuma *et al*., 2008 ^b^ | 19 | 9 | 21.6(1.5) | 23 | Monetary Gambling | | Reward Outcomes | | | | 490 | | | | | NA |  |
| Jiang *et al*., 2014 | 16 | 7 | 22.4(2.28) | 7 | Stimuli matching | | Reward Outcomes | | | | 1399.64 | | | | | NA |  |
| Katahira *et al*., 2015 | 25 | 13 | 24.44(5.28) | 10 | Picture matching | | Reward Outcomes | | | | NA | | | | | NA |  |
| Kim *et al*., 2015 ^a^ | 19 | 10 | 24.47(2.99) | 6 | Monetary reward | | Reward Anticipation | | | | 787.04 | | | | | NA |  |
|  |  |  |  | 6 | Monetary reward | | Reward Outcomes | | | |  | | | | |  |  |
| Knutson *et al*., 2001 ^ab^ | 9 | 2 | 26.45(5.85) | 13 | MID | | Reward Anticipation | | | | 197.09 | | | | | 61 |  |
| Knutson *et al*., 2008 ^ab^ | 12 | 4 | 28.67(4.25) | 8 | MID | | Reward Anticipation | | | | 68 | | | | | 202.81 |  |
|  |  |  |  | 16 | MID | | Reward Outcomes | | | |  | | | | |  |  |
| Koch *et al*., 2014 | 42 | 17 | 25.5(5.2) | 15 | MID | | Reward Outcomes | | | | NA | | | | | NA |  |
| Kurniawan *et al*., 2013 | 19 | 11 | 21.7(2.7) | 3 | Cue prediction | | Reward Outcomes | | | | ~400 | | | | | NA |  |
| Leotti & Delgado, 2014 | 24 | 12 | 21 | 1 | Gambling | | Reward Outcomes | | | | NA | | | | | NA |  |
| Li *et al*., 2016 | 19 | 11 | 19.63(2.14) | 4 | MID | | Reward Anticipation | | | | NA | | | | | NA |  |
| Linke *et al*., 2010 ^b^ | 33 | 17 | 22.64(2.92) | 16 | Pattern recognition | | Reward Outcomes | | | | 700 | | | | | NA |  |
| Macoveanu *et al*., 2016 ^a^ | 29 | 0 | 25.2(5.9) | 13 | Card Guessing | | Reward Risk | | | | NA | | | | | NA |  |
|  |  |  |  | 10 | Card Guessing | | Reward Outcomes | | | |  | | | | |  |  |
| Martin *et al*., 2009 ^b^ | 20 | NA | 19.6(1.35); 18-22 | 2 | Conditioning | | Reward Outcomes | | | | NA | | | | | NA |  |
| Mas-Herrero *et al*., 2015 | 20 | 5 | 22.9(2.9) | 21 | Gambling | | Reward Outcomes | | | | NA | | | | | NA |  |
| Météreau & Dreher, 2013 ^b^ | 20 | 10 | 24.4; 18-33 | 10 | Conditioning | | Reward Outcomes | | | | NA | | | | | 97 |  |
| Montoya *et al*., 2014 | 20 | 20 | 23(3.4); 19-30 | 14 | MID | | Reward Outcomes | | | | 274.26 | | | | | NA |  |
| Mullett & Tunney, 2013 ^a^ | 14 | 5 | 20-27 | 9 | MID | | Reward Outcomes | | | | 202 | | | | | 59 |  |
|  |  |  |  | 22 | MID | | Reward Outcomes | | | |  | | | | |  |  |
| Nieuwenhuis *et al*., 2005 ^b^ | 14 | 8 | 25.4; 22-31 | 10 | Monetary Gambling | | Reward Outcomes | | | | 884 | | | | | NA |  |
| Pascucci *et al*., 2017 | 20 | 6 | 24(3) | 3 | Shooting targets | | Reward Outcomes | | | | NA | | | | | NA |  |
| Petrovic et al., 2008 ^ab^ | 15 | 15 | 24; 20-36 | 12 | Wheel of fortune | | Reward Outcomes | | | | NA | | | | | NA |  |
|  |  |  |  | 14 | Wheel of fortune | | Reward Outcomes | | | |  | | | | |  |  |
| Pfabigan *et al*., 2014 | 25 | 12 | 23.8(3.6) | 12 | MID | | Reward Anticipation | | | | 218.91 | | | | | NA |  |
| Rogers *et al*., 2004 ^ab^ | 14 | 9 | 23.36(1.36) | 12 | Wheel of fortune | | Decision phase | | | | 1970 | | | | | NA |  |
|  |  |  |  | 12 | Wheel of fortune | | Reward Outcomes | | | |  | | | | |  |  |
| Rudorf & Hare, 2014 | 22 | 16 | 22(2.49) | 16 | Binary choice | | Reward Stimuli | | | | NA | | | | | 96.7 |  |
| Ruissen *et al*., 2018 | 19 | 7 | 21.8(2.4) | 4 | “I told you so” | | Reward Outcomes | | | | NA | | | | | 62 |  |
| Saji *et al*., 2013 | 18 | 10 | 29.6(6.94) | 22 | MID | | Reward Anticipation | | | | NA | | | | | NA |  |
| Schouw *et al*., 2013 | 8 | 8 | 22(3) | 2 | MID | | Reward Anticipation | | | | 197.4 | | | | | 56.5 |  |
| Sescousse *et al*., 2010 ^b^ | 18 | 18 | 24(3.3) | 6 | MID | | Reward Outcomes | | | | ~500 | | | | | ~96 |  |
| Sescousse *et al*., 2014 | 38 | 38 | 27.5(6.8) | 28 | MID | | Reward Anticipation | | | | NA | | | | | 97.1 |  |
| Shigemune *et al*., 2014 | 33 | 8 | 20.7; 19-24 | 7 | Gambling | | Reward Outcomes | | | | 1553.2 | | | | | NA |  |
| Simon *et al*., 2015 ^a^ | 27 | 0 | ~26.5 | 7 | MID | | Reward Anticipation | | | | 462 | | | | | 98.1 |  |
|  |  |  |  | 9 | MID | | Reward Outcomes | | | |  | | | | |  |  |
| Smith *et al*., 2011 ^ab^ | 35 | 35 | ~27.8; 18-43 | 10 | Passive viewing | | Reward Outcomes | | | | NA | | | | | 91 |  |
|  |  |  |  | 5 | Passive viewing | | Reward Outcomes | | | |  | | | | |  |  |
| Speer *et al*., 2014 | 19 | 9 | 26.1(7.78) | 11 | Cued recall | | Reward Outcomes | | | | NA | | | | | NA |  |
| Treadway *et al*., 2013 ^a^ | 38 | 20 | 22; 18-34 | 13 | MID | | Reward Anticipation | | | | NA | | | | | NA |  |
|  |  |  |  | 2 | MID | | Reward Outcomes | | | |  | | | | |  |  |
| Vaidya *et al*., 2013 ^ac^ | 18 | 9 | 27.72(1.36); 26-30 | 9 | MID | | Reward Anticipation | | | | 197 | | | | | 67 |  |
|  |  |  |  | 15 | MID | | Reward Outcomes | | | |  | | | | |  |  |
| Van Leijenhorst *et al*., 2010b ^abc^ | 15 | 8 | 20.2(1.6); 18-23 | 3 | Slot machine | | Reward Anticipation | | | | ~1200 | | | | | 70 |  |
|  |  |  |  | 3 | Slot machine | | Reward Outcomes | | | |  | | | | |  |  |
| Varnum *et al*., 2014 | 15 | 5 | 19-24 | 10 | MID | | Reward Outcomes | | | | NA | | | | | NA |  |
| Vermeer *et al*., 2014 ^a^ | 26 | 14 | 22(2.68); 19-27 | 6 | Time estimation | | Reward Outcomes | | | | NA | | | | | NA |  |
|  |  |  |  | 10 | Time estimation | | Reward Risk | | | |  | | | | |  |  |
| Vollm *et al*., 2007 ^b^ | 14 | 14 | 27.1(7.1); 20-44 | 20 | Target detection | | Reward Outcomes | | | | 498.9 | | | | | 99 |  |
| Votinov *et al*., 2015 | 69 | 31 | 23.8(5.4) | 37 | Time estimation | | Reward Outcomes | | | | 198 | | | | | NA |  |
| Weis *et al*., 2013 | 39 | 21 | 24; 18-31 | 25 | Instrumental learning | | Reward Outcomes | | | | 405 | | | | | ~80% |  |
| Wilson *et al*., 2014 | 44 | 24 | 26.1(7); 18-45 | 8 | Smoking lapse | | Reward Outcomes | | | | NA | | | | | NA |  |
| Young & Nusslock, 2016 | 20 | 10 | 21.19(1.34) | 16 | MID | | Reward Anticipation | | | | NA | | | | | NA |  |
|  | | | | | |  | |  |  |  | |  |  |  |  | |  |

Note: n = sample size; R = Right handed; SD = Standard deviation; NA = not available; ^a^ = article includes more than one contrast; ^b^ = article included from previous meta-analysis (Sescousse *et al*., 2013); ^c^ = article included in children/adolescents search; MID = monetary incentive delay task

SM Table 4: Concordant brain regions related to reward outcomes for each age group

| Children |  |  |  |  |  |  |
| --- | --- | --- | --- | --- | --- | --- |
| Cluster # | Volume mm^3^ | ALE Value | x | y | z | Brain region |
| 1 | 720 | 0.021 | -14 | 2 | -8 | L Globus Pallidus |
|  |  |  |  |  |  |  |
| Adolescents |  |  |  |  |  |  |
| Cluster # | Volume mm^3^ | ALE Value | x | y | z | Brain region |
| 1 | 5712 | 0.045 | -10 | 10 | -4 | L Caudate Head |
|  |  | 0.026 | -18 | -6 | -14 | L Amygdala |
| 2 | 5328 | 0.062 | 12 | 10 | -4 | R Putamen |
|  |  | 0.020 | 18 | -8 | -12 | R Amygdala |
|  |  | 0.017 | 26 | 0 | 8 | R Putamen |
|  |  | 0.016 | 16 | 4 | 20 | R Caudate Body |
|  |  | 0.016 | 22 | -2 | 16 | R Putamen |
|  |  | 0.015 | 26 | 14 | -6 | R Claustrum |
| 3 | 1960 | 0.028 | -2 | -40 | 28 | L Posterior Cingulate Gyrus BA 31 |
|  |  | 0.017 | 6 | -30 | 24 | R Posterior Cingulate Gyrus BA 23 |
|  |  | 0.015 | 4 | -24 | 22 | R Posterior Cingulate Gyrus BA 23 |
| 4 | 1248 | 0.022 | 44 | 38 | 22 | R Middle Frontal Gyrus BA 46 |
|  |  | 0.021 | 44 | 32 | 20 | R Middle Frontal Gyrus BA 46 |
|  |  | 0.019 | 42 | 30 | 12 | R Middle Frontal Gyrus BA 46 |
| 5 | 1056 | 0.031 | 0 | 46 | -6 | L Medial Frontal Gyrus BA 10 |
| 6 | 648 | 0.021 | -18 | 2 | 20 | L Caudate Body |
|  |  |  |  |  |  |  |
| Adults |  |  |  |  |  |  |
| Cluster # | Volume mm^3^ | ALE Value | x | y | z | Brain region |
| 1 | 17448 | 0.101 | 10 | 8 | -2 | R Caudate Head |
|  |  | 0.081 | -14 | 8 | -4 | L Putamen |
|  |  | 0.031 | 0 | -14 | 8 | L Thalamus (dorsal medial) |
|  |  | 0.029 | 0 | -6 | 10 | L Thalamus (dorsal medial) |
|  |  | 0.024 | 26 | 18 | -6 | R Claustrum |
|  |  | 0.020 | -28 | -12 | 0 | L Putamen |
| 2 | 5712 | 0.045 | 0 | 40 | 8 | L Anterior Cingulate Gyrus BA 32 |
|  |  | 0.025 | 4 | 30 | 28 | R Anterior Cingulate Gyrus BA 32 |
| 3 | 1568 | 0.041 | -2 | -36 | 32 | L Posterior Cingulate Gyrus BA 31 |
| 4 | 1488 | 0.031 | -4 | -52 | 18 | L Posterior Cingulate Gyrus BA 30 |

| Conjunctions |  | | |  | | | |  | | | |  | | | | | |  | | | | |  | | | | | | |  |  |  |  |
| --- | --- | --- | --- | --- | --- | --- | --- | --- | --- | --- | --- | --- | --- | --- | --- | --- | --- | --- | --- | --- | --- | --- | --- | --- | --- | --- | --- | --- | --- | --- | --- | --- | --- |
| Adolescents-AND-Children | | | | | |  | | | | |  | | | | | |  | | | |  | | | | |  | | |  | | | | |
| Cluster # | Volume mm^3^ | | | ALE Value | | | | x | | | | y | | | | | | z | | | | | Brain region | | | | | | |  |  |  |  |
| 1 | 696 | | | 0.021 | | | | -14 | | | | 2 | | | | | | -8 | | | | | L Globus Pallidus | | | | | | |  |  |  |  |
|  |  | | |  | | | |  | | | |  | | | | | |  | | | | |  | | | | | | |  |  |  |  |
| Adults-AND-Adolescents | | | |  | | | | |  | | | | | |  | | | |  | | | | |  | | | |  | | | | |  |
| Cluster # | Volume mm^3^ | | | ALE Value | | | | x | | | | y | | | | | | z | | | | | Brain region | | | | | | |  |  |  |  |
| 1 | 4208 | | | 0.045 | | | | -10 | | | | 10 | | | | | | 4 | | | | | L Caudate Head | | | | | | |  |  |  |  |
|  |  | | | 0.021 | | | | -18 | | | | -4 | | | | | | -12 | | | | | L Amygdala | | | | | | |  |  |  |  |
| 2 | 4112 | | | 0.062 | | | | 12 | | | | 10 | | | | | | -4 | | | | | R Putamen | | | | | | |  |  |  |  |
|  |  | | | 0.018 | | | | 18 | | | | -6 | | | | | | -12 | | | | | R Amygdala | | | | | | |  |  |  |  |
|  |  | | | 0.015 | | | | 26 | | | | 14 | | | | | | -6 | | | | | R Claustrum | | | | | | |  |  |  |  |
| 3 | 624 | | | 0.027 | | | | -2 | | | | -38 | | | | | | 30 | | | | | L Posterior Cingulate Gyrus BA 31 | | | | | | |  |  |  |  |
| 4 | 496 | | | 0.023 | | | | -2 | | | | 46 | | | | | | -4 | | | | | L Anterior Cingulate Gyrus BA 32 | | | | | | |  |  |  |  |
|  |  | | |  | | | |  | | | |  | | | | | |  | | | | |  | | | | | | |  |  |  |  |
| Adults-AND-Children | | |  | | | |  | | | | | |  | | |  | | | | | |  | | | | |  | | | | |  |  |
| Cluster # | Volume mm^3^ | | | ALE Value | | | | x | | | | y | | | | | | z | | | | | Brain region | | | | | | |  |  |  |  |
| 1 | 720 | | | 0.021 | | | | -14 | | | | 2 | | | | | | -8 | | | | | L Globus Pallidus | | | | | | |  |  |  |  |
|  |  | | |  | | | |  | | | |  | | | | | |  | | | | |  | | | | | | |  |  |  |  |
| Contrasts | | | |  | | | | |  | | | | | |  | | | |  | | | | |  | | | |  | | | | |  |
| Adolescents > Adults | | | |  | | | | |  | | | | | |  | | | |  | | | | |  | | | |  | | | | |  |
| Cluster # | Volume mm^3^ | | | ALE Value | | | | x | | | | y | | | | | | z | | | | | Brain region | | | | | | |  |  |  |  |
| 1 | 664 | | | 3.352 | | | | 40 | | | | 32 | | | | | | 14 | | | | | R Middle Frontal Gyrus BA 46 | | | | | | |  |  |  |  |
|  |  | | | 3.267 | | | | 46 | | | | 36 | | | | | | 22 | | | | | R Middle Frontal Gyrus BA 46 | | | | | | |  |  |  |  |
| 2 | 536 | | | 3.155 | | | | 10 | | | | 14 | | | | | | -2 | | | | | R Caudate Head | | | | | | |  |  |  |  |
|  |  | | | 2.794 | | | | 8 | | | | 16 | | | | | | -6 | | | | | R Caudate Head | | | | | | |  |  |  |  |
| 3 | 528 | | | 3.290 | | | | 2 | | | | -32 | | | | | | 22 | | | | | R Posterior Cingulate Gyrus BA 23 | | | | | | |  |  |  |  |
|  |  | | | 3.035 | | | | -4 | | | | -36 | | | | | | 22 | | | | | L Posterior Cingulate Gyrus BA 23 | | | | | | |  |  |  |  |
|  |  | | | 2.862 | | | | 2 | | | | -36 | | | | | | 22 | | | | | R Posterior Cingulate Gyrus BA 23 | | | | | | |  |  |  |  |
| 4 | 400 | | | 3.290 | | | | -22 | | | | -8 | | | | | | -20 | | | | | L Amygdala | | | | | | |  |  |  |  |
| 5 | 376 | | | 3.155 | | | | -18 | | | | 6 | | | | | | 22 | | | | | L Caudate Body | | | | | | |  |  |  |  |
| 6 | 232 | | | 3.011 | | | | -10 | | | | 14 | | | | | | -6 | | | | | L Caudate Head | | | | | | |  |  |  |  |
|  |  | | |  | | | |  | | | |  | | | | | |  | | | | |  | | | | | | |  |  |  |  |
| Adults > Children | |  | | |  | | | | |  | | | |  | | | | | |  | | | | |  | | | | | |  |  |  |
| Cluster # | Volume mm^3^ | | | ALE Value | | | | x | | | | y | | | | | | z | | | | | Brain region | | | | | | |  |  |  |  |
| 1 | 1784 | | | 3.238 | | | | 15 | | | | 13 | | | | | | 2 | | | | | R Caudate Head | | | | | | |  |  |  |  |
|  |  | | | 2.947 | | | | 18 | | | | 6 | | | | | | 4 | | | | | R Putamen | | | | | | |  |  |  |  |
|  |  | | | 2.726 | | | | 16 | | | | -4 | | | | | | 4 | | | | | R Globus Pallidus (lateral) | | | | | | |  |  |  |  |
|  |  | | | 2.706 | | | | 6 | | | | 8 | | | | | | -2 | | | | | R Caudate Head | | | | | | |  |  |  |  |
|  |  | | | 2.575 | | | | 10 | | | | 2 | | | | | | 10 | | | | | R Caudate Body | | | | | | |  |  |  |  |
| 2 | 1376 | | | 3.238 | | | | -2 | | | | -45.3 | | | | | | 20 | | | | | L Posterior Cingulate Gyrus BA 30 | | | | | | |  |  |  |  |
|  |  | | | 3.194 | | | | 0 | | | | -50 | | | | | | 24 | | | | | L Posterior Cingulate BA 23 | | | | | | |  |  |  |  |
|  |  | | | 3.061 | | | | -4 | | | | -54 | | | | | | 24 | | | | | L Posterior Cingulate BA 31 | | | | | | |  |  |  |  |
|  |  | | | 3.035 | | | | -8 | | | | -50 | | | | | | 22 | | | | | L Posterior Cingulate BA 31 | | | | | | |  |  |  |  |
|  |  | | | 2.847 | | | | 0 | | | | -52 | | | | | | 16 | | | | | L Posterior Cingulate BA 23 | | | | | | |  |  |  |  |
| 3 | 768 | | | 2.929 | | | | 4 | | | | 42 | | | | | | 12 | | | | | R Anterior Cingulate BA 32 | | | | | | |  |  |  |  |
|  |  | | | 2.678 | | | | 2 | | | | 34 | | | | | | 12 | | | | | R Anterior Cingulate BA 24 | | | | | | |  |  |  |  |
| 4 | 592 | | | 3.540 | | | | 4 | | | | -16 | | | | | | 4 | | | | | R Thalamus (dorsal medial) | | | | | | |  |  |  |  |
| 5 | 368 | | | 2.894 | | | | -2 | | | | -34 | | | | | | 26 | | | | | L Posterior Cingulate BA 23 | | | | | | |  |  |  |  |
|  |  | | | 2.820 | | | | 0 | | | | -40 | | | | | | 28 | | | | | L Posterior Cingulate BA 31 | | | | | | |  |  |  |  |
|  |  | | |  | | | |  | | | |  | | | | | |  | | | | |  | | | | | | |  |  |  |  |
| Adolescents > Children | | | | | |  | | | | |  | | | | | |  | | | |  | | | | |  | | |  | | | | |
| Cluster # | Volume mm^3^ | | | ALE Value | | | | x | | | | y | | | | | | z | | | | | Brain region | | | | | | |  |  |  |  |
| 1 | 2568 | | | 3.890 | | | | 11.6 | | | | 16.2 | | | | | | -4.7 | | | | | R Caudate Head | | | | | | |  |  |  |  |
|  |  | | | 3.719 | | | | 7 | | | | 17 | | | | | | 0 | | | | | R Caudate Head | | | | | | |  |  |  |  |
|  |  | | | 3.540 | | | | 14.8 | | | | 10.8 | | | | | | 4.4 | | | | | R Caudate Head | | | | | | |  |  |  |  |
|  |  | | | 2.669 | | | | 24 | | | | 4 | | | | | | 8 | | | | | R Putamen | | | | | | |  |  |  |  |
| 2 | 1752 | | | 3.540 | | | | 2.7 | | | | -28.7 | | | | | | 21.8 | | | | | L Posterior Cingulate BA 23 | | | | | | |  |  |  |  |
|  |  | | | 3.431 | | | | -2 | | | | -40 | | | | | | 20 | | | | | L Posterior Cingulate BA 29 | | | | | | |  |  |  |  |
|  |  | | | 3.290 | | | | -5 | | | | -29 | | | | | | 24 | | | | | L Posterior Cingulate BA 23 | | | | | | |  |  |  |  |
|  |  | | | 3.155 | | | | 2 | | | | -38 | | | | | | 22 | | | | | R Posterior Cingulate BA 23 | | | | | | |  |  |  |  |
|  |  | | | 3.121 | | | | 5.5 | | | | -29.5 | | | | | | 26 | | | | | R Posterior Cingulate BA 23 | | | | | | |  |  |  |  |
|  |  | | | 3.090 | | | | -2.8 | | | | -43.6 | | | | | | 24.4 | | | | | L Posterior Cingulate BA 23 | | | | | | |  |  |  |  |
|  |  | | |  | | | |  | | | |  | | | | | |  | | | | |  | | | | | | |  |  |  |  |
| Adults > Adolescents | | | |  | | | | |  | | | | | |  | | | |  | | | | |  | | | |  | |  |  |  |  |
| no suprathreshold clusters | | | |  | | | | |  | | | | | |  | | | |  | | | | |  | | | |  | |  |  |  |  |
| Children > Adolescents | | | |  | | | | |  | | | | | |  | | | |  | | | | |  | | | |  | |  |  |  |  |
| no suprathreshold clusters | | | |  | | | | |  | | | | | |  | | | |  | | | | |  | | | |  | |  |  |  |  |
| Children > Adults | | | |  | | | | |  | | | | | |  | | | |  | | | | |  | | | |  | |  |  |  |  |
| no suprathreshold clusters | | | |  | | | | |  | | | | | |  | | | |  | | | | |  | | | |  | | | | |  |
|  | | | |  | | | |  | | | |  | | | | | |  | | | | |  | | | | | | |  |  |  |  |

Note: Talairach coordinates (x, y, z) of brain regions surviving a cluster-level threshold of p < 0.05 and a cluster forming threshold of p < 0.01 for single studies. L = Left, R = Right; BA = Brodmann Area, ALE = Activation Likelihood Estimate.

SM Table 5: Concordant brain regions related to reward anticipation

| Cluster # | Volume mm^3^ | ALE Value | x | y | z | Brain region |
| --- | --- | --- | --- | --- | --- | --- |
| 1 | 18376 | 0.081 | -10 | 6 | 2 | L Caudate Head |
|  |  | 0.080 | 10 | 10 | 2 | R Caudate Head |
|  |  | 0.032 | -6 | -24 | -2 | L Red Nucleus |
|  |  | 0.030 | 4 | -8 | 8 | R Thalamus |
|  |  | 0.024 | -14 | -24 | -6 | L Thalamus (medial geniculum) |
|  |  | 0.022 | -20 | -6 | -12 | L Amygdala |
|  |  | 0.021 | 6 | -26 | -2 | R Thalamus |
|  |  | 0.018 | -10 | -16 | 10 | L Thalamus (dorsal medial) |
| 2 | 1216 | 0.039 | -6 | 0 | 50 | L Medial Frontal Gyrus (BA 6) |
|  |  | 0.038 | 2 | 0 | 50 | R Medial Frontal Gyrus (BA 6) |
| 3 | 1144 | 0.048 | 30 | 20 | 4 | R Insula BA 13 |
| 4 | 720 | 0.027 | 30 | -8 | 48 | R Precentral Gyrus (BA 6) |
|  |  |  |  |  |  |  |
|  |  |  |  |  |  |  |

Note: Talairach coordinates (x, y, z) of brain regions surviving a cluster-level threshold of p < 0.05 and a cluster forming threshold of p < 0.01 for single studies. L = Left, R = Right; BA = Brodmann Area, ALE = Activation Likelihood Estimate.

SM Table 6: Concordant brain regions related to Monetary Incentive Delay (MID) task

| Cluster # | Volume mm^3^ | ALE Value | x | y | z | Brain region |
| --- | --- | --- | --- | --- | --- | --- |
| 1 | 20192 | 0.092 | 10 | 10 | -4 | R Caudate Head |
|  |  | 0.088 | -8 | 8 | -2 | L Caudate Head |
|  |  | 0.035 | -8 | -16 | 12 | L Thalamus (dorsal medial) |
|  |  | 0.032 | -6 | -24 | -2 | L Red Nucleus |
|  |  | 0.031 | 0 | -12 | 10 | L Thalamus |
|  |  | 0.029 | -16 | -26 | -4 | L Thalamus (medial geniculum) |
|  |  | 0.027 | 6 | -26 | -2 | R Thalamus |
|  |  | 0.021 | 6 | -16 | -12 | R Substantia Nigra |
|  |  | 0.020 | 16 | -8 | -10 | R Parahippocampal Gyrus (BA 28) |
| 2 | 3944 | 0.045 | 2 | 0 | 50 | R Medial Frontal Gyrus (BA 6) |
|  |  | 0.039 | -4 | 0 | 48 | L Medial Frontal Gyrus (BA 6) |
|  |  | 0.028 | -6 | 8 | 34 | L Cingulate Gyrus (BA 24) |
|  |  | 0.024 | 6 | 8 | 44 | R Medial Frontal Gyrus (BA 32) |
|  |  | 0.022 | 6 | 12 | 32 | R Cingulate Gyrus (BA 24) |
| 3 | 1840 | 0.040 | 30 | 18 | 4 | R Claustrum |
|  |  | 0.021 | 40 | 8 | 4 | R Insula (BA 13) |
| 4 | 1032 | 0.029 | -38 | -20 | 54 | L Precentral Gyrus (BA 4) |
|  |  | 0.026 | -30 | -12 | 44 | L Middle Frontal Gyrus (BA 6) |
| 5 | 960 | 0.032 | 32 | -10 | 48 | R Middle Frontal Gyrus (BA 6) |
| 6 | 944 | 0.029 | 2 | 46 | -8 | R Medial Frontal Gyrus (BA 10) |
| 7 | 736 | 0.025 | -30 | 14 | 10 | L Insula (BA 13) |
|  |  |  |  |  |  |  |
|  |  |  |  |  |  |  |

Note: Talairach coordinates (x, y, z) of brain regions surviving a cluster-level threshold of p < 0.05 and a cluster forming threshold of p < 0.01 for single studies. L = Left, R = Right; BA = Brodmann Area, ALE = Activation Likelihood Estimate.

References

Albrecht, K., Abeler, J., Weber, B., & Falk, A. (2014). The brain correlates of the effects of monetary and verbal rewards on intrinsic motivation. *Front Neurosci*, 8, 303. doi: 10.3389/fnins.2014.00303

Apaydın, N., Üstün, S., Kale, E. H., Çelikağ, İ., Özgüven, H. D., Baskak, B., & Çiçek, M. (2018). Neural Mechanisms Underlying Time Perception and Reward Anticipation. *Front Hum Neurosci*, 12, 115. doi: 10.3389/fnhum.2018.00115

Banich, M. T., De La Vega, A., Andrews-Hanna, J. R., Mackiewicz Seghete, K., Du, Y., & Claus, E. D. (2013). Developmental trends and individual differences in brain systems involved in intertemporal choice during adolescence. *Psychol Addict Behav*, 27(2), 416. doi: 10.1037/a0031991

Barkley-Levenson, E. E., Van Leijenhorst, L., & Galván, A. (2013). Behavioral and neural correlates of loss aversion and risk avoidance in adolescents and adults. *Dev Cogn Neurosci*, 3, 72-83. <https://doi.org/10.1016/j.dcn.2012.09.007>

Barman, A., Richter, S., Soch, J., Deibele, A., Richter, A., Assmann, A., ... & Schott, B. H. (2015). Gender-specific modulation of neural mechanisms underlying social reward processing by Autism Quotient. *Soc Cogn Affect Neurosci*, 10(11), 1537-1547. doi: 10.1093/scan/nsv044

Behan, B., Stone, A., & Garavan, H. (2015). Right prefrontal and ventral striatum interactions underlying impulsive choice and impulsive responding. *Hum Brain Mapp*, 36(1), 187-198. doi: 10.1002/hbm.22621

Bijleveld, E., Custers, R., Van der Stigchel, S., Aarts, H., Pas, P., & Vink, M. (2014). Distinct neural responses to conscious versus unconscious monetary reward cues. *Hum Brain Mapp*, 35(11), 5578-5586. doi: 10.1002/hbm.22571

Bischoff-Grethe, A., Buxton, R. B., Paulus, M. P., Fleisher, A. S., Yang, T. T., & Brown, G. G. (2015). Striatal and pallidal activation during reward modulated movement using a translational paradigm. *J Int Neuropsychol Soc*, 21(6), 399-411. doi: 10.1017/S1355617715000491

Bjork, J. M., Chen, G., Smith, A. R., & Hommer, D. W. (2010a). Incentive elicited mesolimbic activation and externalizing symptomatology in adolescents. *J Child Psychol Psychiatry*, 51(7), 827-837. doi: 10.1111/j.1469-7610.2009.02201.x

Bjork, J. M., Knutson, B., Fong, G. W., Caggiano, D. M., Bennett, S. M., & Hommer, D. W. (2004). Incentive-elicited brain activation in adolescents: similarities and differences from young adults. *J Neurosci*, 24(8), 1793-1802. DOI: 10.1523/JNEUROSCI.4862-03.2004

Bjork, J. M., Knutson, B., & Hommer, D. W. (2008). Incentive elicited striatal activation in adolescent children of alcoholics. *Addiction*, 103(8), 1308-1319. doi: 10.1111/j.1360-0443.2008.02250.x

Bjork, J. M., Smith, A. R., Chen, G., & Hommer, D. W. (2010b). Adolescents, adults and rewards: comparing motivational neurocircuitry recruitment using fMRI. *PloS one*, 5(7), e11440. doi: 10.1371/journal.pone.0011440

Boecker, R., Holz, N. E., Buchmann, A. F., Blomeyer, D., Plichta, M. M., Wolf, I., ... & Laucht, M. (2014). Impact of early life adversity on reward processing in young adults: EEG-fMRI results from a prospective study over 25 years. *PLoS One*, 9(8), e104185. <https://doi.org/10.1371/journal.pone.0104185>

Bolenz, F., Reiter, A. M., & Eppinger, B. (2017). Developmental changes in learning: Computational mechanisms and social influences. *Front Psychol*, 8, 2048.

Bothe, N., Zschucke, E., Dimeo, F., Heinz, A., Wüstenberg, T., & Ströhle, A. (2013). Acute exercise influences reward processing in highly trained and untrained men. *Med Sci Sports Exerc*, 45(3), 583-591. doi: 10.1249/MSS.0b013e318275306f

Brevers, D., Noël, X., He, Q., Melrose, J. A., & Bechara, A. (2016). Increased ventral‐striatal activity during monetary decision making is a marker of problem poker gambling severity. *Addict Biol*, 21(3), 688-699. doi: 10.1111/adb.12239

Camara, E., Rodriguez-Fornells, A., & Münte, T. F. (2009). Functional connectivity of reward processing in the brain. *Front Human Neurosci*, 2, 19. doi: 10.3389/neuro.09.019.2008

Canessa, N., Crespi, C., Motterlini, M., Baud-Bovy, G., Chierchia, G., Pantaleo, G., ... & Cappa, S. F. (2013). The functional and structural neural basis of individual differences in loss aversion. *J Neurosci*, 33(36), 14307-14317. DOI: <https://doi.org/10.1523/JNEUROSCI.0497-13.2013>

Choi, J. M., Padmala, S., Spechler, P., & Pessoa, L. (2014). Pervasive competition between threat and reward in the brain. *Soc Cogn Affect Neurosci*, 9(6), 737-750. DOI: 10.1093/scan/nst053

Clark, L., Lawrence, A. J., Astley-Jones, F., & Gray, N. (2009). Gambling near-misses enhance motivation to gamble and recruit win-related brain circuitry. *Neuron*, 61(3), 481-490. doi: 10.1016/j.neuron.2008.12.031. doi: 10.1038/nn.2558

Cohen, J. R., Asarnow, R. F., Sabb, F. W., Bilder, R. M., Bookheimer, S. Y., Knowlton, B. J., & Poldrack, R. A. (2010). A unique adolescent response to reward prediction errors. *Nat Neurosci*, 13(6), 669. doi: 10.1038/nn.2558

Costumero, V., Barrós-Loscertales, A., Fuentes, P., Rosell-Negre, P., Bustamante, J. C., & Ávila, C. (2016). BAS-drive trait modulates dorsomedial striatum activity during reward response-outcome associations. *Brain imaging Behav*, 10(3), 869-879. DOI: 10.1007/s11682-015-9466-5

Cox, S. M., Andrade, A., & Johnsrude, I. S. (2005). Learning to like: a role for human orbitofrontal cortex in conditioned reward. *J Neurosci*, 25(10), 2733-2740. DOI: 10.1523/JNEUROSCI.3360-04.2005

Crowley, T. J., Dalwani, M. S., Mikulich-Gilbertson, S. K., Du, Y. P., Lejuez, C. W., Raymond, K. M., & Banich, M. T. (2010). Risky decisions and their consequences: neural processing by boys with antisocial substance disorder. *PloS one*, 5(9), e12835. doi: 10.1371/journal.pone.0012835

Crowley, T. J., Dalwani, M. S., Sakai, J. T., Raymond, K. M., McWilliams, S. K., Banich, M. T., & Mikulich-Gilbertson, S. K. (2017). Children’s brain activation during risky decision-making: A contributor to substance problems? *Drug Alcohol Depend*, 178, 57-65. doi: 10.1016/j.drugalcdep.2017.02.028

Cservenka, A., & Nagel, B. J. (2012). Risky decision‐making: an FMRI study of youth at high risk for alcoholism. *Alcohol Clin Exp Res*, 36(4), 604-615. doi: 10.1111/j.1530-0277.2011.01650.x

de Macks, Z. A. O., Bunge, S. A., Bell, O. N., Wilbrecht, L., Kriegsfeld, L. J., Kayser, A. S., & Dahl, R. E. (2016). Risky decision-making in adolescent girls: The role of pubertal hormones and reward circuitry. *Psychoneuroendocrinology*, 74, 77-91. doi: 10.1016/j.psyneuen.2016.08.013

Delgado‐Rico, E., Soriano‐Mas, C., Verdejo‐Román, J., S. Río‐Valle, J., & Verdejo‐García, A. (2013). Decreased insular and increased midbrain activations during decision‐making under risk in adolescents with excess weight. *Obesity*, 21(8), 1662-1668. doi: 10.1002/oby.20375

Dong, G., Lin, X., Zhou, H., & Du, X. (2014). Decision-making after continuous wins or losses in a randomized guessing task: implications for how the prior selection results affect subsequent decision-making. *Behav Brain Funct*, 10(1), 11. doi: 10.1186/1744-9081-10-11

Edmiston, E. K., Merkle, K., & Corbett, B. A. (2015). Neural and cortisol responses during play with human and computer partners in children with autism. *Soc Cogn Affect Neurosci*, 10(8), 1074-1083. doi: 10.1093/scan/nsu159

Elliott, R., Newman, J. L., Longe, O. A., & Deakin, J. W. (2003). Differential response patterns in the striatum and orbitofrontal cortex to financial reward in humans: a parametric functional magnetic resonance imaging study. *J Neurosci*, 23(1), 303-307

Ernst, M., Nelson, E. E., Jazbec, S., McClure, E. B., Monk, C. S., Leibenluft, E., ... & Pine, D. S. (2005). Amygdala and nucleus accumbens in responses to receipt and omission of gains in adults and adolescents. *Neuroimage*, 25(4), 1279-1291. DOI: 10.1016/j.neuroimage.2004.12.038

Ferdinand, N. K., & Opitz, B. (2014). Different aspects of performance feedback engage different brain areas: Disentangling valence and expectancy in feedback processing. *Sci Rep*, 4, 5986. <https://doi.org/10.1038/srep05986>

Forbes, E. E., Ryan, N. D., Phillips, M. L., Manuck, S. B., Worthman, C. M., Moyles, D. L., ... & Dahl, R. E. (2010). Healthy adolescents' neural response to reward: associations with puberty, positive affect, and depressive symptoms. *J Am Acad Child Adolesc Psychiatry*, 49(2), 162-172

Fujiwara, J., Tobler, P. N., Taira, M., Iijima, T., & Tsutsui, K. I. (2009). Segregated and integrated coding of reward and punishment in the cingulate cortex. *J Neurophysiol*, 101(6), 3284-3293. DOI: 10.1152/jn.90909.2008

Funayama, T., Ikeda, Y., Tateno, A., Takahashi, H., Okubo, Y., Fukayama, H., & Suzuki, H. (2014). Modafinil augments brain activation associated with reward anticipation in the nucleus accumbens. *Psychopharmacology*, 231(16), 3217-3228. DOI: 10.1007/s00213-014-3499-0

Gleich, T., Lorenz, R. C., Pöhland, L., Raufelder, D., Deserno, L., Beck, A., ... & Gallinat, J. (2015). Frontal glutamate and reward processing in adolescence and adulthood. *Brain Struct Func*, 220(6), 3087-3099. doi: 10.1007/s00429-014-0844-3

Goerlich, K. S., Votinov, M., Lammertz, S. E., Winkler, L., Spreckelmeyer, K. N., Habel, U., ... & Gossen, A. (2017). Effects of alexithymia and empathy on the neural processing of social and monetary rewards. *Brain Struct Func*, 222(5), 2235-2250. doi: 10.1007/s00429-016-1339-1

Gossen, A., Groppe, S. E., Winkler, L., Kohls, G., Herrington, J., Schultz, R. T., ... & Spreckelmeyer, K. N. (2013). Neural evidence for an association between social proficiency and sensitivity to social reward. *Soc Cogn Affect Neurosci*, 9(5), 661-670. doi: 10.1093/scan/nst033

Hardin, M. G., Pine, D. S., & Ernst, M. (2009). The influence of context valence in the neural coding of monetary outcomes. *Neuroimage*, 48(1), 249-257. doi: 10.1016/j.neuroimage.2009.06.050

Helfinstein, S. M., Benson, B., Perez-Edgar, K., Bar-Haim, Y., Detloff, A., Pine, D. S., ... & Ernst, M. (2011). Striatal responses to negative monetary outcomes differ between temperamentally inhibited and non-inhibited adolescents. *Neuropsychologia*, 49(3), 479-485. DOI: 10.1016/j.neuropsychologia.2010.12.015

Hu, J., Lee, D., Hu, S., Zhang, S., Chao, H., & Chiang-shan, R. L. (2016). Individual variation in the neural processes of motor decisions in the stop signal task: the influence of novelty seeking and harm avoidance personality traits. *Brain Struct Func*, 221(5), 2607-2618. doi: 10.1007/s00429-015-1061-4

Ivanov, I., Liu, X., Shulz, K., Fan, J., London, E., Friston, K., ... & Newcorn, J. H. (2012). Parental substance abuse and function of the motivation and behavioral inhibition systems in drug-naïve youth. *Psychiatry Res Neuroimaging*, 201(2), 128-135. doi: 10.1016/j.pscychresns.2011.08.004

Izuma, K., Saito, D. N., & Sadato, N. (2008). Processing of social and monetary rewards in the human striatum. *Neuron*, 58(2), 284-294. doi: 10.1016/j.neuron.2008.03.020

Jiang, Y., Kim, S. I., & Bong, M. (2014). Effects of reward contingencies on brain activation during feedback processing. *Front Hum Neurosci*, 8, 656. doi: 10.3389/fnhum.2014.00656

Joseph, J. E., Zhu, X., Lynam, D., & Kelly, T. H. (2016). Modulation of meso-limbic reward processing by motivational tendencies in young adolescents and adults. *Neuroimage*, 129, 40-54. doi: 10.1016/j.neuroimage.2015.12.005

Kappel, V., Koch, A., Lorenz, R. C., Brühl, R., Renneberg, B., Lehmkuhl, U., ... & Beck, A. (2013). CID: a valid incentive delay paradigm for children. *J Neural Transm*, 120(8), 1259-1270. doi: 10.1007/s00702-012-0962-0

Katahira, K., Matsuda, Y. T., Fujimura, T., Ueno, K., Asamizuya, T., Suzuki, C., ... & Okada, M. (2015). Neural basis of decision making guided by emotional outcomes. *J Neurophysiol*, 113(9), 3056-3068. doi: 10.1152/jn.00564.2014

Kim, J. E., Son, J. W., Choi, W. H., Kim, Y. R., Oh, J. H., Lee, S., & Kim, J. K. (2014). Neural responses to various rewards and feedback in the brains of adolescent Internet addicts detected by functional magnetic resonance imaging. *Psychiatry Clin Neurosci*, 68(6), 463-470. doi: 10.1111/pcn.12154

Kim, S. H., Yoon, H., Kim, H., & Hamann, S. (2015). Individual differences in sensitivity to reward and punishment and neural activity during reward and avoidance learning. *Soc Cogn Affect Neurosci*, 10(9), 1219-1227. doi: 10.1093/scan/nsv007

Knutson, B., Adams, C. M., Fong, G. W., & Hommer, D. (2001). Anticipation of increasing monetary reward selectively recruits nucleus accumbens. *J Neurosci*, *21*(16), RC159-RC159

Knutson, B., & Greer, S. M. (2008). Anticipatory affect: neural correlates and consequences for choice. *Philos Trans R Soc Lond B Biol Sci*, 363(1511), 3771-3786. doi: 10.1098/rstb.2008.0155

Koch, K., Wagner, G., Schachtzabel, C., Schultz, C. C., Güllmar, D., Reichenbach, J. R., ... & Schlösser, R. G. (2014). Association between white matter fiber structure and reward‐related reactivity of the ventral striatum. *Hum Brain Mapp*, 35(4), 1469-1476. doi: 10.1002/hbm.22284

Kohls, G., Thönessen, H., Bartley, G. K., Grossheinrich, N., Fink, G. R., Herpertz-Dahlmann, B., & Konrad, K. (2014). Differentiating neural reward responsiveness in autism versus ADHD. *Dev Cogn Neurosci*, 10, 104-116. doi: 10.1016/j.dcn.2014.08.003

Kurniawan, I. T., Guitart-Masip, M., Dayan, P., & Dolan, R. J. (2013). Effort and valuation in the brain: the effects of anticipation and execution. *J Neurosci*, 33(14), 6160-6169. doi: 10.1523/JNEUROSCI.4777-12.2013

Lahat, A., Benson, B. E., Pine, D. S., Fox, N. A., & Ernst, M. (2018). Neural responses to reward in childhood: relations to early behavioral inhibition and social anxiety. *Soc Cogn Affect Neurosci*, 13(3), 281-289. DOI: 10.1093/scan/nsw122

Leotti, L. A., & Delgado, M. R. (2014). The value of exercising control over monetary gains and losses. *Psychol Sci*, 25(2), 596-604. doi: 10.1177/0956797613514589

Li, X., Li, Z., Li, K., Zeng, Y. W., Shi, H. S., Xie, W. L., ... & Chan, R. C. (2016). The neural transfer effect of working memory training to enhance hedonic processing in individuals with social anhedonia. *Sci Rep*, 6, 35481. doi: 10.1038/srep35481

Linke, J., Kirsch, P., King, A. V., Gass, A., Hennerici, M. G., Bongers, A., & Wessa, M. (2010). Motivational orientation modulates the neural response to reward. *Neuroimage*, 49(3), 2618-2625. <https://doi.org/10.1016/j.neuroimage.2009.09.013>

Macoveanu, J., Henningsson, S., Pinborg, A., Jensen, P., Knudsen, G. M., Frokjaer, V. G., & Siebner, H. R. (2016). Sex-steroid hormone manipulation reduces brain response to reward. *Neuropsychopharmacology*, 41(4), 1057. doi: 10.1038/npp.2015.236

Martin, L. E., Potts, G. F., Burton, P. C., & Montague, P. R. (2009). Electrophysiological and hemodynamic responses to reward prediction violation. *Neuroreport*, 20(13), 1140. doi: 10.1097/WNR.0b013e32832f0dca

Mas-Herrero, E., Ripollés, P., HajiHosseini, A., Rodríguez-Fornells, A., & Marco-Pallarés, J. (2015). Beta oscillations and reward processing: coupling oscillatory activity and hemodynamic responses. *Neuroimage*, 119, 13-19. doi: 10.1016/j.neuroimage.2015.05.095

Météreau, E., & Dreher, J. C. (2013). Cerebral correlates of salient prediction error for different rewards and punishments. *Cereb Cortex*, 23(2), 477-487. DOI: 10.1093/cercor/bhs037

Montoya, E. R., Bos, P. A., Terburg, D., Rosenberger, L. A., & van Honk, J. (2014). Cortisol administration induces global down-regulation of the brain's reward circuitry. *Psychoneuroendocrinology*, 47, 31-42. doi: 10.1016/j.psyneuen.2014.04.022

Mullett, T. L., & Tunney, R. J. (2013). Value representations by rank order in a distributed network of varying context dependency. *Brain Cogn*, 82(1), 76-83. DOI: 10.1016/j.bandc.2013.02.010

Navas, J. F., Barrós-Loscertales, A., Costumero-Ramos, V., Verdejo-Román, J., Vilar-López, R., & Verdejo-García, A. (2018). Excessive body fat linked to blunted somatosensory cortex response to general reward in adolescents. *Int J Obes*, 42(1), 88. doi: 10.1038/ijo.2017.207

Nieuwenhuis, S., Heslenfeld, D. J., von Geusau, N. J. A., Mars, R. B., Holroyd, C. B., & Yeung, N. (2005). Activity in human reward-sensitive brain areas is strongly context dependent. *Neuroimage*, 25(4), 1302-1309

Paloyelis, Y., Mehta, M. A., Faraone, S. V., Asherson, P., & Kuntsi, J. (2012). Striatal sensitivity during reward processing in attention-deficit/hyperactivity disorder. *J Am Acad Child Adolesc Psychiatry*, 51(7), 722-732. doi: 10.1016/j.jaac.2012.05.006

Pascucci, D., Hickey, C., Jovicich, J., & Turatto, M. (2017). Independent circuits in basal ganglia and cortex for the processing of reward and precision feedback. *Neuroimage*, 162, 56-64. doi: 10.1016/j.neuroimage.2017.08.067

Paulsen, D., Carter, R. M., Platt, M., Huettel, S. A., & Brannon, E. M. (2012). Neurocognitive development of risk aversion from early childhood to adulthood. *Front Hum Neurosci*, 5, 178. doi: 10.3389/fnhum.2011.00178

Peters, J., Bromberg, U., Schneider, S., Brassen, S., Menz, M., Banaschewski, T., ... & Heinz, A. (2011). Lower ventral striatal activation during reward anticipation in adolescent smokers. *Am J Psychiatry*, 168(5), 540-549. https://doi.org/10.1176/appi.ajp.2010.10071024

Petrovic, P., Pleger, B., Seymour, B., Klöppel, S., De Martino, B., Critchley, H., & Dolan, R. J. (2008). Blocking central opiate function modulates hedonic impact and anterior cingulate response to rewards and losses. *J Neurosci*, 28(42), 10509-10516. DOI:10.1523/JNEUROSCI.2807-08.2008

Pfabigan, D. M., Seidel, E. M., Sladky, R., Hahn, A., Paul, K., Grahl, A., ... & Windischberger, C. (2014). P300 amplitude variation is related to ventral striatum BOLD response during gain and loss anticipation: an EEG and fMRI experiment. *Neuroimage*, 96, 12-21. doi: 10.1016/j.neuroimage.2014.03.077

Ripke, S., Hübner, T., Mennigen, E., Müller, K. U., Rodehacke, S., Schmidt, D., ... & Smolka, M. N. (2012). Reward processing and intertemporal decision making in adults and adolescents: the role of impulsivity and decision consistency. *Brain Res*, 1478, 36-47. doi: 10.1016/j.brainres.2012.08.034

Rogers, R. D., Ramnani, N., Mackay, C., Wilson, J. L., Jezzard, P., Carter, C. S., & Smith, S. M. (2004). Distinct portions of anterior cingulate cortex and medial prefrontal cortex are activated by reward processing in separable phases of decision-making cognition. *Biol Psychiatry*, 55(6), 594-602. DOI: 10.1016/j.biopsych.2003.11.012

Rudorf, S., & Hare, T. A. (2014). Interactions between dorsolateral and ventromedial prefrontal cortex underlie context-dependent stimulus valuation in goal-directed choice. *J Neurosci*, 34(48), 15988-15996. doi: 10.1523/JNEUROSCI.3192-14.2014

Ruissen, M. I., Overgaauw, S., & de Bruijn, E. R. A. (2018). Being right, but losing money: the role of striatum in joint decision making. *Sci Rep*, 8, 6711. <https://doi.org/10.1038/s41598-018-24617-3>

Saji, K., Ikeda, Y., Kim, W., Shingai, Y., Tateno, A., Takahashi, H., ... & Suzuki, H. (2013). Acute NK1 receptor antagonist administration affects reward incentive anticipation processing in healthy volunteers. *Int J Neuropsychopharmacol*, 16(7), 1461-1471. doi: 10.1017/S1461145712001678

Scheres, A., Milham, M. P., Knutson, B., & Castellanos, F. X. (2007). Ventral striatal hyporesponsiveness during reward anticipation in attention-deficit/hyperactivity disorder. *Biol Psychiatry*, 61(5), 720-724. DOI: 10.1016/j.biopsych.2006.04.042

Schlund, M. W., Siegle, G. J., Ladouceur, C. D., Silk, J. S., Cataldo, M. F., Forbes, E. E., ... & Ryan, N. D. (2010). Nothing to fear? Neural systems supporting avoidance behavior in healthy youths. *Neuroimage*, 52(2), 710-719. doi: 10.1016/j.neuroimage.2010.04.244

Schneider, S., Brassen, S., Bromberg, U., Banaschewski, T., Conrod, P., Flor, H., ... & Nees, F. (2012). Maternal interpersonal affiliation is associated with adolescents’ brain structure and reward processing. *Transl Psychiatry*, 2(11), e182. doi: 10.1038/tp.2012.113

Schouw, M. L. J., De Ruiter, M. B., Kaag, A. M., van den Brink, W., Lindauer, R. J. L., & Reneman, L. (2013). Dopaminergic dysfunction in abstinent dexamphetamine users: results from a pharmacological fMRI study using a reward anticipation task and a methylphenidate challenge. *Drug Alcohol Depend*, 130(1-3), 52-60. doi: 10.1016/j.drugalcdep.2012.10.010

Schwenck, C., Ciaramidaro, A., Selivanova, M., Tournay, J., Freitag, C. M., & Siniatchkin, M. (2017). Neural correlates of affective empathy and reinforcement learning in boys with conduct problems: fMRI evidence from a gambling task. *Behav Brain Res*, 320, 75-84. doi: 10.1016/j.bbr.2016.11.037

Scott‐Van Zeeland, A. A., Dapretto, M., Ghahremani, D. G., Poldrack, R. A., & Bookheimer, S. Y. (2010). Reward processing in autism. *Autism Res*, 3(2), 53-67. doi: 10.1002/aur.122

Sescousse, G., Li, Y., & Dreher, J. C. (2014). A common currency for the computation of motivational values in the human striatum. *Soc Cogn Affect Neurosci*, 10(4), 467-473. doi: 10.1093/scan/nsu074

Sescousse, G., Redouté, J., & Dreher, J. C. (2010). The architecture of reward value coding in the human orbitofrontal cortex. *J Neurosci*, 30(39), 13095-13104. DOI:10.1523/JNEUROSCI.3501-10.2010

Shigemune, Y., Tsukiura, T., Kambara, T., & Kawashima, R. (2013). Remembering with gains and losses: effects of monetary reward and punishment on successful encoding activation of source memories. *Cereb Cortex*, 24(5), 1319-1331. DOI: 10.1093/cercor/bhs415

Simon, J. J., Skunde, M., Wu, M., Schnell, K., Herpertz, S. C., Bendszus, M., ... & Friederich, H. C. (2015). Neural dissociation of food-and money-related reward processing using an abstract incentive delay task. *Soc Cogn Affect Neurosci*, 10(8), 1113-1120. doi: 10.1093/scan/nsu162

Smith, AB., Halari, R., Giampetro, V., Brammer, M., Rubia, K. (2011). Developmental effects of reward on sustained attention networks. Neuroimage. 56(3):1693-1704. doi:10.1016/j.neuroimage.2011.01.072

Speer, M. E., Bhanji, J. P., & Delgado, M. R. (2014). Savoring the past: positive memories evoke value representations in the striatum. *Neuron*, 84(4), 847-856. doi: 10.1016/j.neuron.2014.09.028

Steinbeis, N., Haushofer, J., Fehr, E., & Singer, T. (2014). Development of behavioral control and associated vmPFC–DLPFC connectivity explains children's increased resistance to temptation in intertemporal choice. *Cereb Cortex*, 26(1), 32-42. doi: 10.1093/cercor/bhu167

Telzer, E. H., Fuligni, A. J., Lieberman, M. D., & Galván, A. (2013). Meaningful family relationships: neurocognitive buffers of adolescent risk taking. *J Cogn Neurosci*, 25(3), 374-387. doi: 10.1162/jocn_a_00331

Thomason, M. E., & Marusak, H. A. (2017). Within-subject neural reactivity to reward and threat is inverted in young adolescents. *Psychol Med*, 47(9), 1549-1560. doi: 10.1017/S0033291716003111

Treadway, M. T., Buckholtz, J. W., & Zald, D. (2013). Perceived stress predicts altered reward and loss feedback processing in medial prefrontal cortex. *Front Hum Neurosci*, 7, 180. doi: 10.3389/fnhum.2013.00180

Vaidya, J. G., Knutson, B., O'Leary, D. S., Block, R. I., & Magnotta, V. (2013). Neural sensitivity to absolute and relative anticipated reward in adolescents. *PLoS One*, 8(3), e58708. <https://doi.org/10.1371/journal.pone.0058708>

van den Bos, W., Crone, E. A., & Güroğlu, B. (2012). Brain function during probabilistic learning in relation to IQ and level of education. *Dev Cogn Neurosci*, 2, S78-S89. doi: 10.1016/j.dcn.2011.09.007

van Duijvenvoorde, A. C., de Macks, Z. A. O., Overgaauw, S., Moor, B. G., Dahl, R. E., & Crone, E. A. (2014). A cross-sectional and longitudinal analysis of reward-related brain activation: effects of age, pubertal stage, and reward sensitivity. *Brain Cogn*, 89, 3-14. <https://doi.org/10.1016/j.bandc.2013.10.005>

van Leijenhorst, L., Crone, E. A., & Bunge, S. A. (2006). Neural correlates of developmental differences in risk estimation and feedback processing. *Neuropsychologia*, 44(11), 2158-2170. DOI: 10.1016/j.neuropsychologia.2006.02.002

van Leijenhorst, L., Moor, B. G., de Macks, Z. A. O., Rombouts, S. A., Westenberg, P. M., & Crone, E. A. (2010a). Adolescent risky decision-making: neurocognitive development of reward and control regions. *Neuroimage*, 51(1), 345-355. doi: 10.1016/j.neuroimage.2010.02.038

van Leijenhorst, L., Zanolie, K., Van Meel, C. S., Westenberg, P. M., Rombouts, S. A., & Crone, E. A. (2010b). What motivates the adolescent? Brain regions mediating reward sensitivity across adolescence. *Cereb Cortex*, 20(1), 61-69. doi: 10.1093/cercor/bhp078

Varnum, M. E., Shi, Z., Chen, A., Qiu, J., & Han, S. (2014). When “Your” reward is the same as “My” reward: Self-construal priming shifts neural responses to own vs. friends' rewards. *Neuroimage*, 87, 164-169. doi: 10.1016/j.neuroimage.2013.10.042

Vermeer, A. B. L., Boksem, M. A., & Sanfey, A. G. (2014). Neural mechanisms underlying context-dependent shifts in risk preferences. *Neuroimage*, 103, 355-363. <https://doi.org/10.1016/j.neuroimage.2014.09.054>

Völlm, B., Richardson, P, McKie, S., Elliott, R., Dolan, M., Deakin, B. (2007). Neuronal correlates of reward and loss in Cluster B personality disorders: a functional magnetic resonance imaging study. Psychiatry Res, 156(2):151-167. doi:10.1016/j.pscychresns.2007.04.008

Votinov, M., Pripfl, J., Windischberger, C., Sailer, U., & Lamm, C. (2015). Better you lose than I do: neural networks involved in winning and losing in a real time strictly competitive game. *Sci Rep*, 5, 11017. <https://doi-org.libproxy1.nus.edu.sg/10.1038/srep11017>

Weis, T., Brechmann, A., Puschmann, S., & Thiel, C. M. (2013). Feedback that confirms reward expectation triggers auditory cortex activity. *J Neurophysiol*, 110(8), 1860-1868. doi: 10.1152/jn.00128.2013

Wiggins, J. L., Schwartz, K. T., Kryza-Lacombe, M., Spechler, P. A., Blankenship, S. L., & Dougherty, L. R. (2017). Neural reactivity to reward in school-age offspring of depressed mothers. *J Affect Disord*, 214, 81-88. doi: 10.1016/j.jad.2017.03.020

Wilson, S. J., Delgado, M. R., McKee, S. A., Grigson, P. S., MacLean, R. R., Nichols, T. T., & Henry, S. L. (2014). Weak ventral striatal responses to monetary outcomes predict an unwillingness to resist cigarette smoking. *Cogn Affect Behav Neurosci*, 14(4), 1196-1207. doi: 10.3758/s13415-014-0285-8

Yaxley, R. H., Van Voorhees, E. E., Bergman, S., Hooper, S. R., Huettel, S. A., & De Bellis, M. D. (2011). Behavioral risk elicits selective activation of the executive system in adolescents: clinical implications. *Front Psychiatry*, 2, 68. doi: 10.3389/fpsyt.2011.00068

Young, C. B., & Nusslock, R. (2016). Positive mood enhances reward-related neural activity. *Soc Cogn Affect Neurosci*, 11(6), 934-944. doi: 10.1093/scan/nsw012
